# Chemical Reactivity and Optical and Pharmacokinetics Studies of 14 Multikinase Inhibitors and Their Docking Interactions Toward ACK1 for Precision Oncology

**DOI:** 10.3389/fchem.2022.843642

**Published:** 2022-04-14

**Authors:** Ruby Srivastava

**Affiliations:** CSIR-Centre for Cellular and Molecular Biology, Hyderabad, India

**Keywords:** inhibitors, ACK1, drug likeliness, docking, cancers

## Abstract

Activated Cdc42-associated kinase 1 (ACK1/TNK2) has a significant role in cell endocytosis, survival, proliferation, and migration. Mutations in ACK1 are closely associated with the occurrence and development of cancers. In this work, a conceptual density functional theory (CDFT)-based computational peptidology (CDFT-CP) method is used to study the chemical reactivity of 14 multikinase inhibitors. Optical properties of these inhibitors are studied by time-dependent density functional theory (TDDFT). Various biological and pharmacokinetic parameters are studied by Osiris, Molinspiration, and BOILED-Egg in SwissADME software tools. Physicochemical and biopharmaceutical (PCB), *Salmonella typhimurium* reverse mutation assay (AMES) mutagenicity, toxicity, and risk prediction are estimated by Simulations plus ADMET Predictor 10.2 software. MD simulations for an active model of ACK1 is carried out by the CABS-flex 2.0 web server, and potential binding pockets for ACK1 are searched using the PrankWeb server. SwissTargetPrediction is used to predict the potential targets for the multikinase inhibitors. Docking studies are carried out for ACK1–multikinase inhibitors using Autodock 4.2 software. Noncovalent interactions for ACK1–multikinase inhibitor complexes are studied using the Protein–Ligand Interaction Profiler (PLIP) server. Results indicated higher binding affinities and strong noncovalent interactions in ACK1–multikinase inhibitor complexes.

## Introduction

Activated Cdc42-associated tyrosine kinase 1 (ACK1) is a member of the VIII tyrosine kinase family (Manser et al., 1993; Lin et al., 2012). The overexpression of ACK1 has a potential impact on other types of diseases also, but recently research studies are mainly focused on its driving effect on cancers (Wang A. et al., 2021). ACK1 can integrate signals of various receptor tyrosine kinases (RTKs), can transfer extracellular signals from receptor tyrosine kinases to cytoplasmic and nuclear effectors, and in turn may regulate the expression levels of some RTKs (Mahajan et al., 2010; Mahajan and Mahajan 2010; Fox et al., 2019) Previous studies indicated that ACK1 regulates the activity of androgen receptor (AR) by tyrosine phosphorylation to increase the growth of hormone-refractory prostate cancers (Mahajan et al., 2005; Mahajan et al., 2012; Mahajan and Mahajan, 2013; Mahajan and Mahajan, 2015). Next-generation sequencing (NGS) studies suggested that recurrent ACK1 gene amplification and somatic mutations in different types of cancers lead to neoplastic transformation. ACK1 is an epigenetic regulator (Wang A. et al., 2021), and its coding gene *TNK2* is related to hematological malignancies and other types of cancers. Since epigenetics is a reversible process, targeting ACK1 could reverse the epigenetic changes and the existence of drug resistance in malignancies. Recently, various approaches, such as fragment-based drug design, repurposing, skeleton transition, and high-throughput screening, have been used to design, discover, and synthesize several highly efficient and specific inhibitors to target ACK1. Reports indicated that 71 FDA-approved small-molecule kinase inhibitors (SMKIs) and additional 16 SMKIs are approved by other authorities. The clinical trials of SMKIs showed that approximately 110 novel kinases are currently being explored as targets. Many SMKIs are in clinical development for diseases other than cancers (Attwood et al., 2021).

The physiological role of ACK1 is involved in human brains (La Torre et al., 2013), inflammation (Zhao et al., 2018), and the immune system. To date, the *ACK1* gene is seen in 131 missense mutations, 39 nonsense mutations, and three fusion mutations within domains of ACK1 in 21 types of cancers (Prieto-Echague et al., 2010; Maxson et al., 2016; Wang Z.-Z. et al., 2021). The latest trends in the inhibitor design include allosteric and covalent inhibitors, bifunctional inhibitors, and chemical degraders (Attwood et al., 2021). FDA has approved many inhibitors which have good therapeutic effects and are used for multiple targets. These multikinase inhibitors can provide potential strategies for simultaneously targeting ACK1 and other targets.

In this study, 14 multikinase inhibitors, namely, GNF-7 (1), (2), (3), (4), dasatinib (5), bosutinib (6), ceritinib (7), PD158780 (8), vemurafenib (9), ADZ9291 (10), sunitinib (11), flavopiridol (12), and gefitinib (13), (14) are selected (Wang A. et al., 2021). Some of these inhibitors have entered clinical trials, and others are approved by FDA as therapeutics for other targets. GNK-7 (1) is used to double-target ACK1 for the NRAS-mutated acute myeloid leukemia cells (Choi et al., 2010; Cho et al., 2018). Complex (2) has a better impact on Ba/F3-NRAS-G12D cells, while Complex (3) exhibits good pharmacokinetic properties. Complex (4) shows inhibitory activities toward Ba/F3-NRAS-G12D and OCI-acute myeloid leukemia (AML3) stability (Wang Z.-Z. et al., 2021). The reference Complex dasatinib (5) (Lombardo et al., 2004) and Complex bosutinib (6) (Stansfield et al., 2013) are Abelson leukemia virus (ABL) and proto-oncogene tyrosine-protein kinase SRC (SRC) kinase inhibitors. Ceritinib (7) is an anaplastic lymphoma kinase (ALK) fusion protein inhibitor (Verduzco et al., 2018). PD158780 (8) is an adenosine 5′-triphosphate (ATP) competitive epidermal growth factor receptor (EGFR) and ACK1 inhibitor (Nur-E-Kamal et al., 2005), while vemurafenib (9) is a potent inhibitor targeting BRAF (V600E). Zelboraf (10) showed selectivity toward several non-rapidly accelerated fibrosarcoma (RAF) kinases (Bollag et al., 2010). Sunitinib (11), flavopiridol (12), and gefitinib (13) are identified as ACK1 inhibitors (Phatak and Zhang, 2013). Complex (14) showed inhibitory activity toward 26 kinases. (Wang A. et al., 2021). As experimental studies and clinical trials are expensive and time-consuming, computational approaches help to predict the chemical and biological properties of complexes and their binding affinities to targets in a better manner. Dasatinib (BMS-354825 or Sprycel) and AIM-100 (Liu et al., 2010; Mahajan et al., 2014) were initially assessed as inhibitors for ACK1 signaling *in vitro* and *in vivo*. Due to its multitarget activity against many tyrosine kinases, dasatinib (5) is selected as a reference inhibitor in this work. The chemical structures of these 14 multikinase inhibitors are given in Supplementary Figure 1. The main objective of this work was to find out which inhibitors would have the best characteristics as a drug candidate.

The density functional theory has been an effective method to predict drug–target interactions in recent years. The conceptual density functional theory (CDFT), developed by Prof. R.G. Parr et al. (Parr and Yang 1989; von Szentpaly 2000; von Szentpaly 2017; von Szentpaly 2018a; von Szentpaly 2018b; von Szentpaly 2020; Kohn et al., 1996; Ayers and Yang 2003; Parr and Yang 1995; Sarkar and Chattaraj 2021; Chattaraj et al., 2002; Chattaraj et al., 2003; Parr et al., 1999), has been used to study the molecular descriptors. The validity, physical basis, and limitations of these descriptors (Kaya and Kaya 2015a; Kaya and Kaya 2015b; Von Szentpály et al., 2020) are being investigated in recent years, and they provide deep insights into the reactivity of the complexes. Few parameters from CDFT-based computational peptidology (CDFT-CP) developed by *Prof. Glossmann Mitnik* et al (Frau et al., 2018; Flores-Holguín et al., 2019a; Flores-Holguín et al., 2019b; Frau et al., 2019; Flores-Holguín et al., 2020a; Flores-Holguín et al. 2020b; Flores-Holguín et al. 2020c; Flores-Holguín et al. 2021) has been incorporated to analyze the physicochemical parameters of these multikinase inhibitors. Previous results related to global descriptors have shown useful predictions for new drug entities (NCEs) (Srivastava, 2021a) and also for drug-like small complexes (Srivastava, 2021b).

## Materials and Methods

**FIGURE 1:**
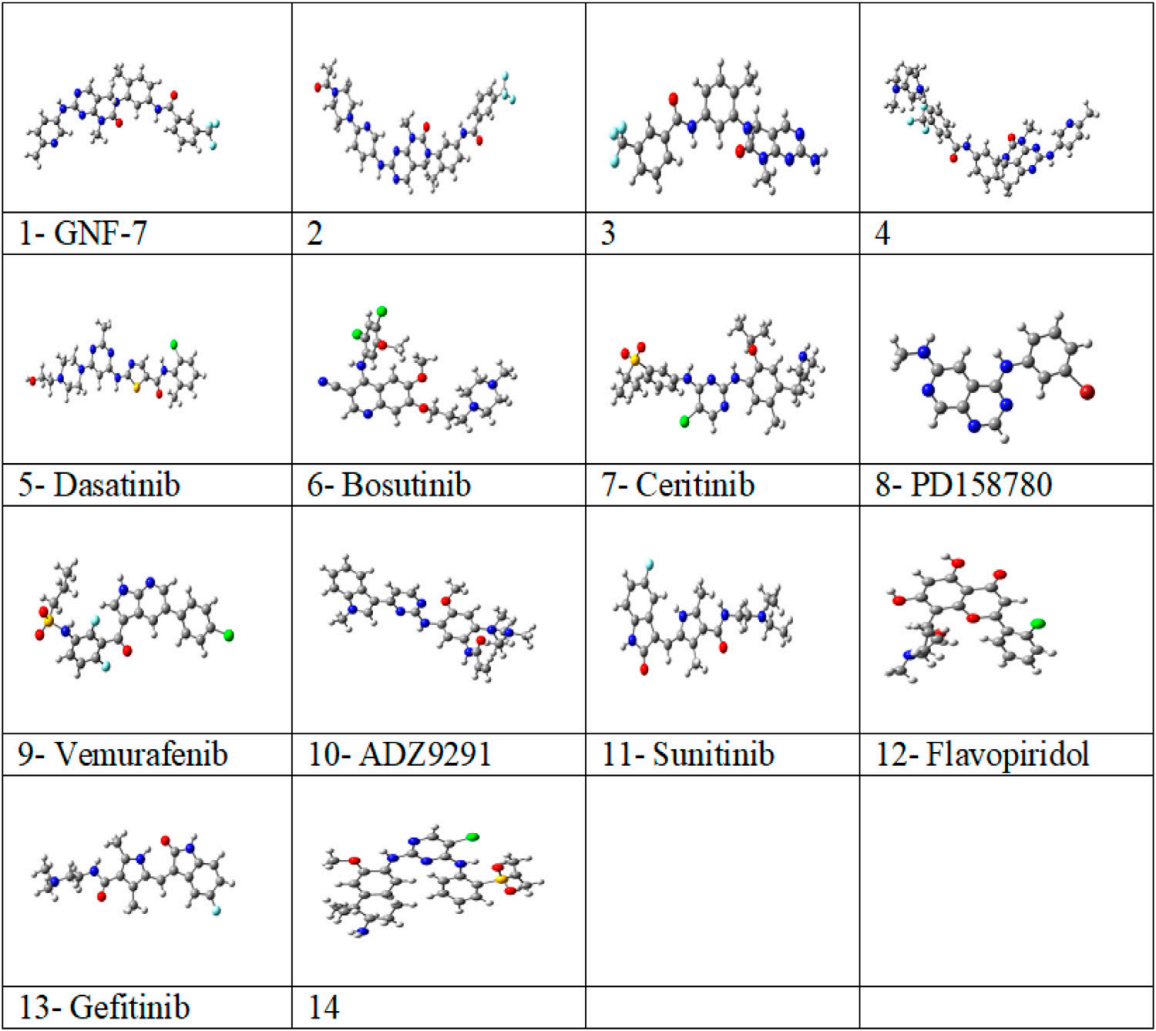
Optimized structures of studied 14 multikinase inhibitors with M06-2X/6-31G (d, p) in solvent (water) from the G16 software program.

The structures of inhibitors have been optimized with M06-2X (Zhao and Truhlar, 2008)/6-31G (d,p) basis sets from G16 software suites (Frisch et al., 2016) with the PCM (Marenich et al., 2009) solvent model. Different conformational structures are optimized, and then the structure with lower minima is selected for further studies. Vibrational frequency analysis is carried out to confirm that there is no negative frequency for the lower minima structures. The optimized structures of the multikinase inhibitors are given in Figure 1. GaussView is used to draw the optimized structures for the inhibitors (Dennington et al., 2003). The TDDFT calculations are carried out on the ground state optimized structures with M06-2X/6-31G (d,p) basis sets using water as a solvent. GaussSum is used to visualize the absorption wavelength, oscillatory strength, and the number of transitions (O’boyle et al., 2008).

The output (pdb) structures are used to generate the SMILES notation for the inhibitors from the online SMILES translator and structure file generator tool, which is given in Table 1.

**TABLE 1:** Common names, identifiers, and simplified molecular input line entry system (SMILES) notation for 14 multikinase inhibitors.

SN	Inhibitor	SMILES notation
1	GNF-7 CAS 839706-07-9	CN1C(=O)N(CC2 = CN = C(NC3 = CC = C(C)N=C3)N=C12)C4 = C(C)C=CC(=C4)NC(=O)C5 = CC(=CC = C5)C(F) (F)F
2	—	CN1C(=O)N(CC2 = CN = C(NC3 = CC = C(N=C3)N4CCN(CC4)C(C) = O)N=C12)C5 = C(C)C=CC(=C5)NC(=O)C6 = CC(=CC = C6)C(F) (F)F
3	—	CN1C(=O)N(CC2 = CN = C(N)N=C12)C3 = C(C)C=CC(=C3)NC(=O)C4 = CC(=CC = C4)C(F) (F)F
4	—	CN(C)C1 = CN(CC1)CC2 = CC = C(C=C2C(F) (F)F)C (=O)NC3 = CC(=C(C)C=C3)N4CC5 = CN = C(NC6 = CC = C(C)N=C6)N=C5N(C)C4 = O
5	Dasatinib DB01254	CC1 = NC(=CC(=N1)N2CCN(CCO)CC2)NC3 = NC = C(S3)C (=O)NC4 = C(C)C=CC = C4Cl
6	Bosutinib DB06616	COC1 = C(Cl)C=C(Cl)C (=C1)NC2 = C3C = C(OC)C (=CC3 = NC = C2C#N)OCCCN4CCN(C)CC4
7	Ceritinib DB09063	CC(C)OC1 = CC(=C(C)C=C1NC2 = NC(=C(Cl)C=N2)NC3 = CC(=CC = C3)[S](=O) (=O)C(C)C)C4CCNCC4
8	PD158780 CAS 171179-06-9	CNC1 = CC2 = C(NC3 = CC = CC(=C3)Br)N=CN = C2C = N1
9	Vemurafenib DB08881	CCC [S](=O) (=O)NC1 = C(F)C (=C(F)C=C1)C (=O)C2 = C [NH]C3 = C2C = C(C=N3)C4 = CC = C(Cl)C=C4
10	ADZ9291 DB09330	COC1 = CC(=C (NC(=O)C=C)C=C1NC2 = NC = CC(=N2)C3 = C [N](C)C4 = C3C = CC = C4)N(C)CCN(C)C
11	Sunitinib DB01268	CCN(CC)CCNC(=O)C1 = C(C)[NH]C (=C1C)CC2 = C(O)[NH]C3 = C2C = C(F)C=C3
12	Flavopiridol DB03496	CN1CCC(C(O)C1)C2 = C3OC(=CC(=O)C3 = C(O)C=C2O)C4 = CC = CC = C4Cl
13	Gefitinib DB00317	CN(C)CCNC(=O)C1 = C(C)[NH]C (=C1C)CC2 = C(O)[NH]C3 = C2C = C(F)C=C3
14	—	COC1C = C2C(CCC2(N)C(C)C)C=C1NC3 = NC = C(Cl)C (=N3)NC4 = CC = CC = C4 [S](=O) (=O)C(C)C

Various molecular properties and bioactivity scores of inhibitors are calculated by Molinspiration Chemoinformatics tools (https://www.molinspiration.com/). (accessed, December 2021) The pharmacokinetic parameters such as mutagenic effect, irritant effect, tumorigenicity, and effect on the reproductive system are estimated by Osiris Property Explorer, 2021 (www.organicchemistry.org/prog/peo/). Other toxicity parameters are predicted by BOILED-Egg in Swiss ADME (Diana and Zoete 2016). Simulations Plus ADMET Predictor (Simulations Plus Inc, 2022) is used to predict the physicochemical and biopharmaceutical (PCB), *Salmonella typhimurium* reverse mutation assay (AMES) mutagenicity, toxicity, and risk prediction. The online SwissTargetPrediction tool is used to predict efficient protein targets for these drug complexes (Diana et al., 2019).

**FIGURE 2:**
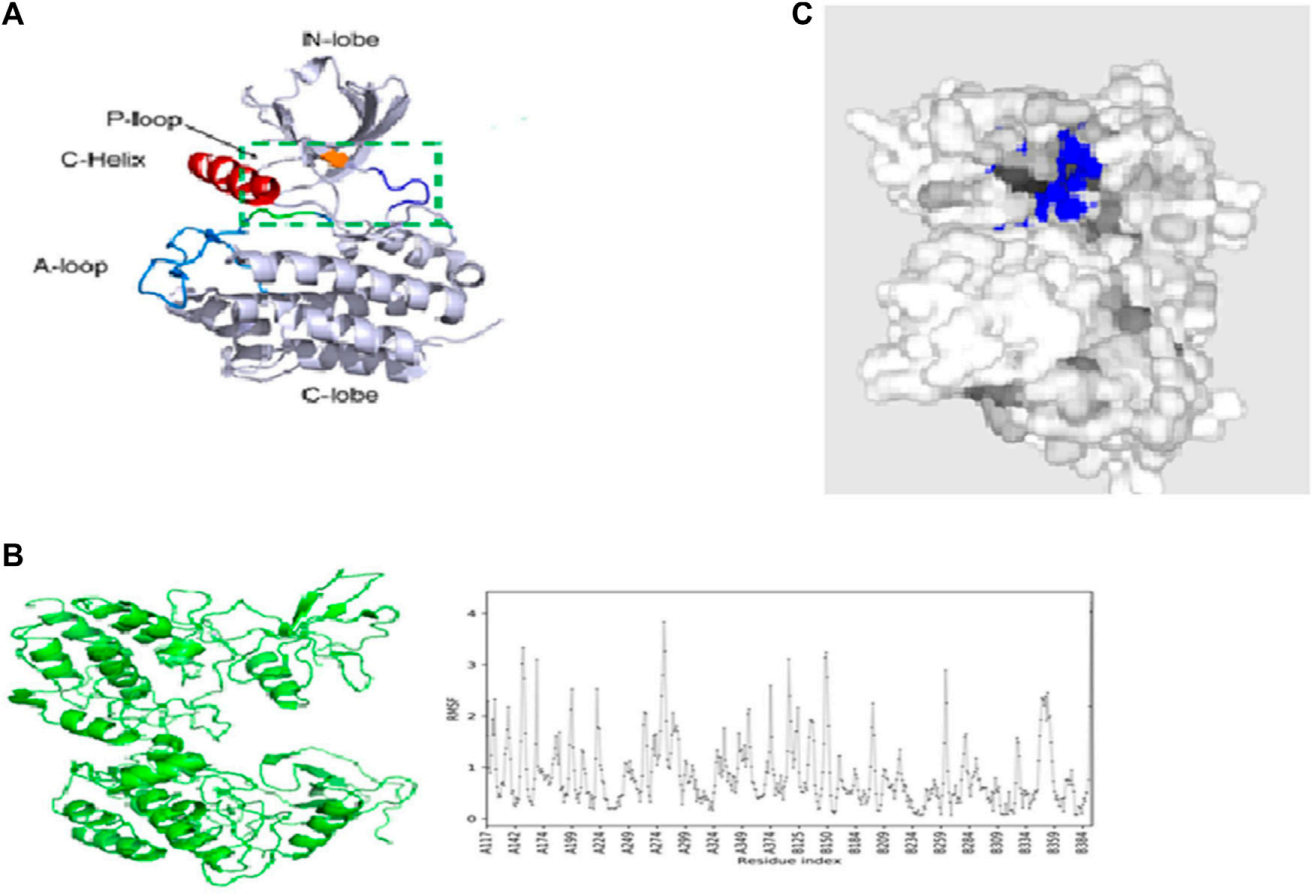
**(A)** Crystal structure of the ACK1 protein (PDB code: 6VQM); **(B)** best model of ACK1 from MD simulations with the RMSF graph (CABS-flex 2.0 server); **(C)** best binding pockets (blue color) of ACK1 as predicted by the Prankweb server.

The structure of ACK1 (pdb id-6VQM) is optimized with the MMF4 force field using Avogadro software (Hanwell et al., 2016), as shown in Figure 2A. Various conformers have been taken for ACK1 structures, and MD simulations are carried out for the lower conformational structure using CABS-flex 2.0 (Kuriata et al., 2018) coarse-grained protein modeling tools. The protein near-native dynamics were obtained from 10 nanosecond MD simulations (all-atom, explicit water, and for all protein metafolds with force fields). Figure 2B shows the best MD-simulated ACK1 model with the root-mean-square fluctuation (RMSF) graph. RMSF measures the average deviation of protein residues over time from the reference values. The PrankWeb server (Jendele et al., 2019) is used to identify potential binding pockets (Pitsillou et al., 2020) for ACK1, which is shown in Figure 2C.

Docking studies for ACK1–inhibitors are carried out by Autodock 4.2.6 packages (Morris et al., 2009). The empirical free energy function and the Lamarckian genetic algorithm were used with other default parameters. Among the top ten binding poses, the model with lower interacting binding energies was selected to study protein–ligand binding interactions by protein–ligand binding profiler (PLIP) online tools (Salentin et al., 2015).

## Results

DFT-based molecular descriptors are numerical characterizations of structural features of complexes. The description of the preferred sites provides a firm explanation for the reactivity of the complexes. The formulas used to calculate the global descriptors are given in Supplementary Information. Global reactivity descriptors and highest occupied molecular orbital (HOMO) and lowest unoccupied molecular orbital (LUMO) values are given in Table 2. The ionization potential (IP) values of all studied complexes are larger, which reflects the ability to lose electrons more easily. All complexes have IP values (4.39–7.62 eV) except Complex 14 (4.11 eV). Electron affinity (EA) reflects the ability of an atom to gain electrons which is lower (0.06–2.4 eV) for most of the complexes. The chemical hardness correlates to the stability, and the reactivity of these inhibitors is measured by the softness. The higher electrophilicity index represents the more reactive nature of these inhibitors, i.e., (1.70–5.53) eV. HOMO and LUMO values of complexes are predictive measures of their interaction with the target complexes. The higher HOMO energy reflected more reactive molecules in the reactions with electrophiles, while lower LUMO energy is necessary for molecular reactions with nucleophiles. The smaller HOMO–LUMO energy gap for these inhibitors corresponds to better stability. Comparing the hardness and HOMO–LUMO gap results, it can be anticipated that hardness can be considered an exact measure, while HOMO–LUMO gap can be taken as an approximation for the inhibitors.

**TABLE 2:** Calculated [ionization energy (lP), electron affinity (EA), and global reactivity descriptors; electronegativity (χ), global hardness (η), global softness (S), and global electrophilicity index (ω)] in eV and highest occupied molecular orbital (HOMO) and lowest unoccupied molecular orbital (LUMO) (au) for 14 multikinase inhibitors.

Complex	IP	EA	χ	η	S	ω	HOMO	LUMO
1	6.94	0.25	3.47	0.13	7.94	0.0159	−0.2084	−0.0584
2	6.48	0.25	3.24	0.13	7.94	0.0159	−0.1893	−0.0586
3	7.25	0.09	3.63	0.05	21.28	0.0022	−0.2183	−0.0573
4	6.14	0.20	3.07	0.10	10.00	0.0100	−0.1620	−0.0631
5	7.01	0.07	3.51	0.03	30.77	0.0011	−0.2132	−0.0538
6	7.12	0.69	3.56	0.35	2.88	0.1204	−0.2172	−0.0767
7	6.62	0.14	3.31	0.07	14.60	0.0047	−0.1946	−0.0448
8	6.96	0.25	3.48	0.12	8.03	0.0155	−0.2014	−0.0662
9	7.62	0.49	3.81	0.24	4.10	0.0595	−0.2306	−0.0699
10	6.06	0.39	3.03	0.20	5.09	0.0386	−0.1771	−0.0294
11	6.63	0.54	3.32	0.27	3.70	0.0732	−0.1975	−0.0733
12	7.14	0.06	3.57	0.03	32.79	0.0009	−0.2085	−0.0519
13	6.64	0.57	3.32	0.29	3.50	0.0818	−0.1961	−0.0740
14	4.39	2.34	2.19	1.17	0.85	1.3701	−0.1358	−0.0245

The wavelength (nm), oscillatory strength, and number of transitions for these multikinase inhibitors are given in Table 3. The TDDFT results showed that the absorption spectra of these complexes lie in the region (246–361 nm), except for Complex 4, which has an absorption wavelength in the visible region (541.35 nm). The percentage for HOMO→LUMO transitions is higher for all inhibitors, which indicates that the interaction between HOMO→ LUMO is enough to lower the energy of the state below one of the HOMO→LUMO states. All DFT results have positive correlations toward the corresponding results of the reference complex dasatinib (5).

**TABLE 3:** Wavelength (nm), oscillatory strength (*f*), and number of transitions for 14 multikinase inhibitors.

S. No.	Wavelength (nm)	Oscillatory strength (f)	Number of transitions
1	275.56	0.0042	H-2→L (86%)
2	300.27	0.7467	H→L+4 (74%)
3	311.68	0.2285	H→L (85%)
4	541.35	0.0545	H→L (98%)
5	319.70	0.8208	H→L (98%)
6	355.84	0.188	H→L (93%)
7	295.54	0.8901	H→L (92%)
8	282.16	0.2783	H→L+1 (58%)
9	246.06	0.0255	H→L+2 (68%)
10	301.39	0.4702	H-1→L+1 (90%)
11	346.71	0.4378	H-2→L (82%)
12	259.17	0.1453	H-2→L (71%)
13	360.55	0.6038	H-2→L (63%)
14	354	0.0654	H-l→L (78%)

The molecular parameters such as clog P, topological polar surface area (TPSA), hydrogen bond donors (HBD), hydrogen bond acceptors (HBA), and molecular weight (MW) are given in Table 4. For orally active drugs, Lipinski’s ‘‘rule of five” states that 1) molecular weight (MW) < 500; 2) the calculated octanol/water partition coefficient (clogP) < 5; 3) there were fewer than five hydrogen bond donors (HBD) (OH and NH groups); and 4) there are less than ten hydrogen bond acceptors (HBA) (notably N and O) (Lipinski et al., 1997). clogP are >5 for complexes 4, 9, and 14, while complexes 2 and 4 have HBA values >10. All the complexes have HBD <5. For the drug-like hits based on the Muegge (Bayer) criteria (Muegge et al., 2001) (200 ≤ MW ≤ 600, −2 ≤ LogP ≤ 5, TPSA ≤ 150, HBD ≤ 5, HBA ≤ 10, and RotB ≤ 15), only complexes 2 and 4 have violation for HBA > 10, so they cannot be considered drug-like hits. It is known that more violations of the Lipinski rules lead to bioavailability problems. The drug-likeliness, drug score, mutagenicity, tumorigenicity, irritant effect, and reproductive effect are given in Table 5. The result indicates that Complex 5 has high toxicity for irritant and reproductive effects. Complex 6 has high toxicity for mutagenicity, tumorigenicity, and reproductive effect, while Complex 7 shows high irritant effect. Complexes 9 and 10 have higher mutagenic effect, while Complex 11 has higher irritant effect. Complex 10 has mild toxicity effect for tumorigenicity, irritant effect, and reproductive effect.

**TABLE 4:** CLP: clog P, TPSA: topological polar surface area, Natoms: number of atoms, MW: molecular weight, S: solubility, HBA: number of hydrogen bond acceptor, and HBD: number of hydrogen bond donor for 14 multikinase inhibitors.

Complex	CLP	TPSA	Natoms	MW	Solubility	HBA	HBD
—	≤5	—	—	<500	—	<10	<5
1	4.88	103.35	40	547	−7.41	9	2
2	4.52	126.89	48	659.67	−7.45	12	2
3	3.39	104.45	33	456.43	−6.34	8	3
4	5.08	109.82	49	671.73	−7.4	11	2
5	3.13	106.5	33	488.02	−4.7	9	3
6	4.98	82.89	36	530.46	−5.45	8	1
7	5.76	105.24	38	558.15	−7.93	8	3
8	3.54	62.73	20	330.19	−4.84	5	2
9	5.56	91.92	33	489.93	−9.33	6	2
10	4.16	78.76	37	497.6	−4.45	9	1
11	2.78	84.15	29	400.5	−4.18	6	4
12	3.46	94.13	28	401.85	−3.87	6	3
13	2.02	84.15	27	352.44	−3.58	6	4
14	5.83	119.24	36	532.11	−6.66	8	4

**TABLE 5:** DL: drug-likeliness, DS: drug score, MUT: mutagenicity, TUMO: tumorigenicity, IRRI: irritant effect, and REPO: reproductive effect for 14 multikinase inhibitors. Green color indicates a low toxic potential, yellow color means mild toxicity, and red color indicates a high probability of toxicity.

Complex	DL	DS	MUT	TUMO	IRRI	REPO
1	−9.21	0.14	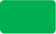	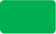	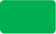	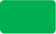
2	−6.19	0.12	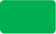	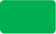	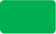	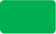
3	−9.22	0.23	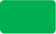	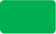	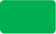	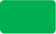
4	−4.0	0.12	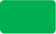	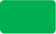	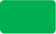	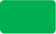
5	8.74	0.2	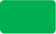	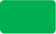	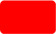	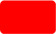
6	4.57	0.06	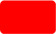	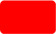	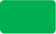	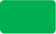
7	−4.07	0.07	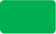	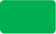	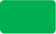	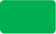
8	−4.4	0.35	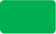	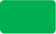	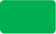	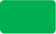
9	−1.11	0.12	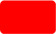	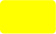	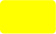	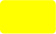
10	−4.58	0.09	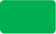	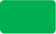	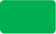	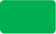
11	−8.72	0.42	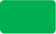	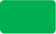	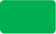	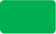
12	6.97	0.7	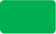	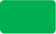	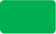	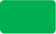
13	5.69	0.8	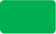	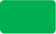	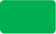	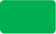
14	−3.64	0.18	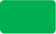	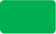	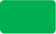	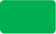

The Biopharmaceutics Classification System (BCS) has defined five criteria for the permeability of drugs: 1) absolute bioavailability or mass balance studies in humans, 2) urinary recovery of unchanged drug in humans, 3) *in vivo* intestinal perfusion studies in humans, 4) *in vitro* permeation studies across a monolayer of cultured epithelial cells, and/or 5) high metabolism as defined under Biopharmaceutical Drug Disposition Classification System (BDDCS) (Ku 2008; Chen et al., 2011). Few of these parameters are given in Table 6, which are predicted by BOILED-Egg in Swiss ADME. Most of the studied complexes showed an inhibitory effect for multitargets. All the inhibitors have Log *K*_p_ values that lie in the range of (−6.0 to 7.0). No BBB permeability is predicted for all inhibitors except Complex 8. The GI absorption is higher for complexes 3, 5, 6, 8, and 10–13.

**TABLE 6:** Pharmacokinetic properties of 14 multikinase inhibitors from BOILED-Egg in Swiss ADME software tools.

S.No.	GI absorption	BBB per	P-gp substrate	CYP1A2 inhibitor	CYP2C19 inhibitor	CYP2C9 inhibitor	CYP2D6 inhibitor	CYP3A4 inhibitor	Log *K*_p_
1	Low	No	No	No	Yes	Yes	Yes	Yes	−6.57
2	Low	No	Yes	No	Yes	Yes	Yes	Yes	−7.73
3	High	No	Yes	No	No	Yes	No	Yes	−7.12
4	Low	No	No	No	Yes	Yes	Yes	Yes	−7.07
5	High	No	No	No	Yes	Yes	Yes	Yes	−6.73
6	High	No	No	No	Yes	Yes	No	No	−5.72
7	Low	No	Yes	No	Yes	No	Yes	No	−5.54
8	High	Yes	No	Yes	Yes	No	Yes	Yes	−5.94
9	Low	No	No	No	Yes	Yes	No	Yes	−5.76
10	High	No	Yes	No	Yes	Yes	Yes	Yes	−6.71
11	High	No	Yes	Yes	Yes	No	Yes	Yes	−6.12
12	High	No	Yes	No	No	No	Yes	Yes	−6.44
13	High	No	Yes	Yes	No	No	Yes	No	−6.48
14	Low	No	Yes	No	Yes	No	No	Yes	−6.39

**FIGURE 3:**
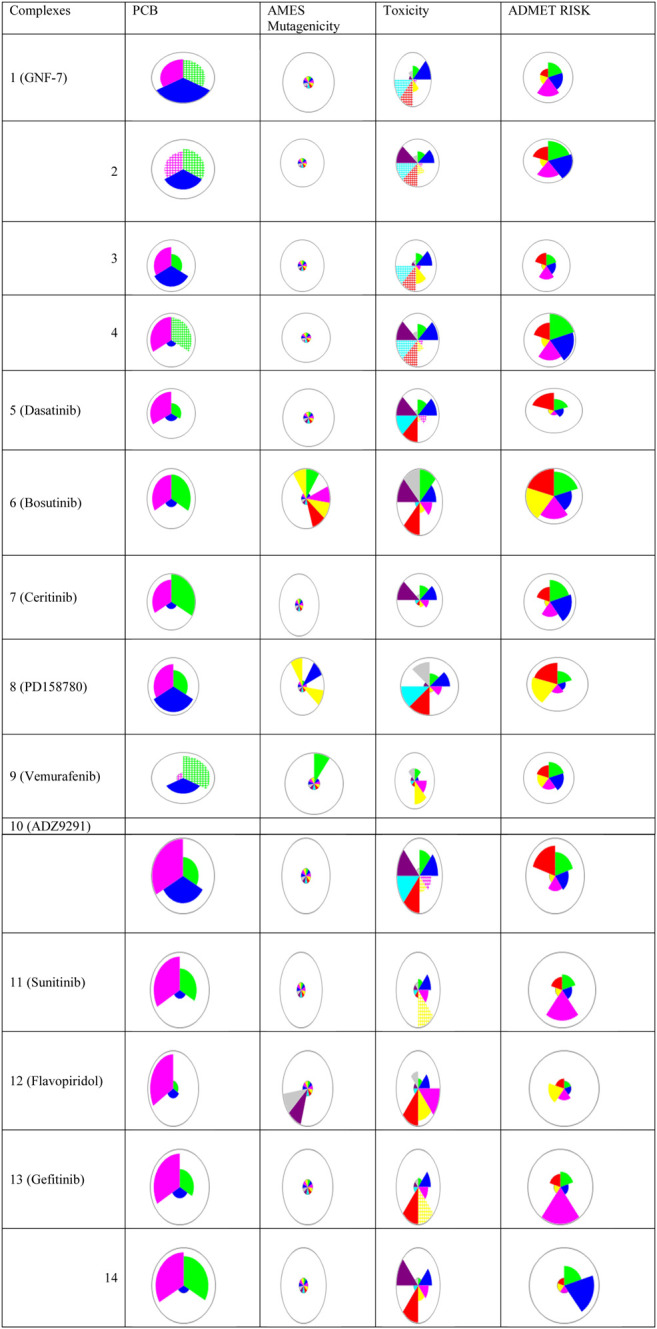
Chemical structures, names, and star plots of physicochemical and biopharmaceutical (PCB), *Salmonella typhimurium* reverse mutation assay (AMES) mutagenicity, toxicity, and risk prediction for 14 multikinase inhibitors as predicted by Simulations Plus ADMET Predictor 10.2. Wedges represent out-of-scope predictions (hatched due to the model’s applicability domain). PCB [solubility + log P (pink), molecular weight (MW) (green), and number of free rotations (blue)]; AMES (colors representing mutagenicity); toxicity (Ser_ALT (blue), Ser_AST (red), herG IC_50_ (green), mutagenic risk (gray), and Ser_LDH (violet)); ADMET risk (toxicity (red); ADMET (green); absorption (blue); CYP risk (pink), and mutagenic risk (yellow)).

The PCB, toxicity, and risk prediction from Simulations Plus ADMET Predictor shows varied PCM, ADMET, and risk prediction for all the inhibitors, as shown in Figure 3. The transport and metabolic properties of these inhibitors are given in Supplementary Figure 4 and Supplementary Figure 5, respectively. All complexes showed good inhibitory activities, but variation in absorption, distribution, metabolism, excretion, toxicity (ADMET), biopharmaceutical properties, and risk prediction indicate that these inhibitors may be used for combination therapies.

**FIGURE 4:**
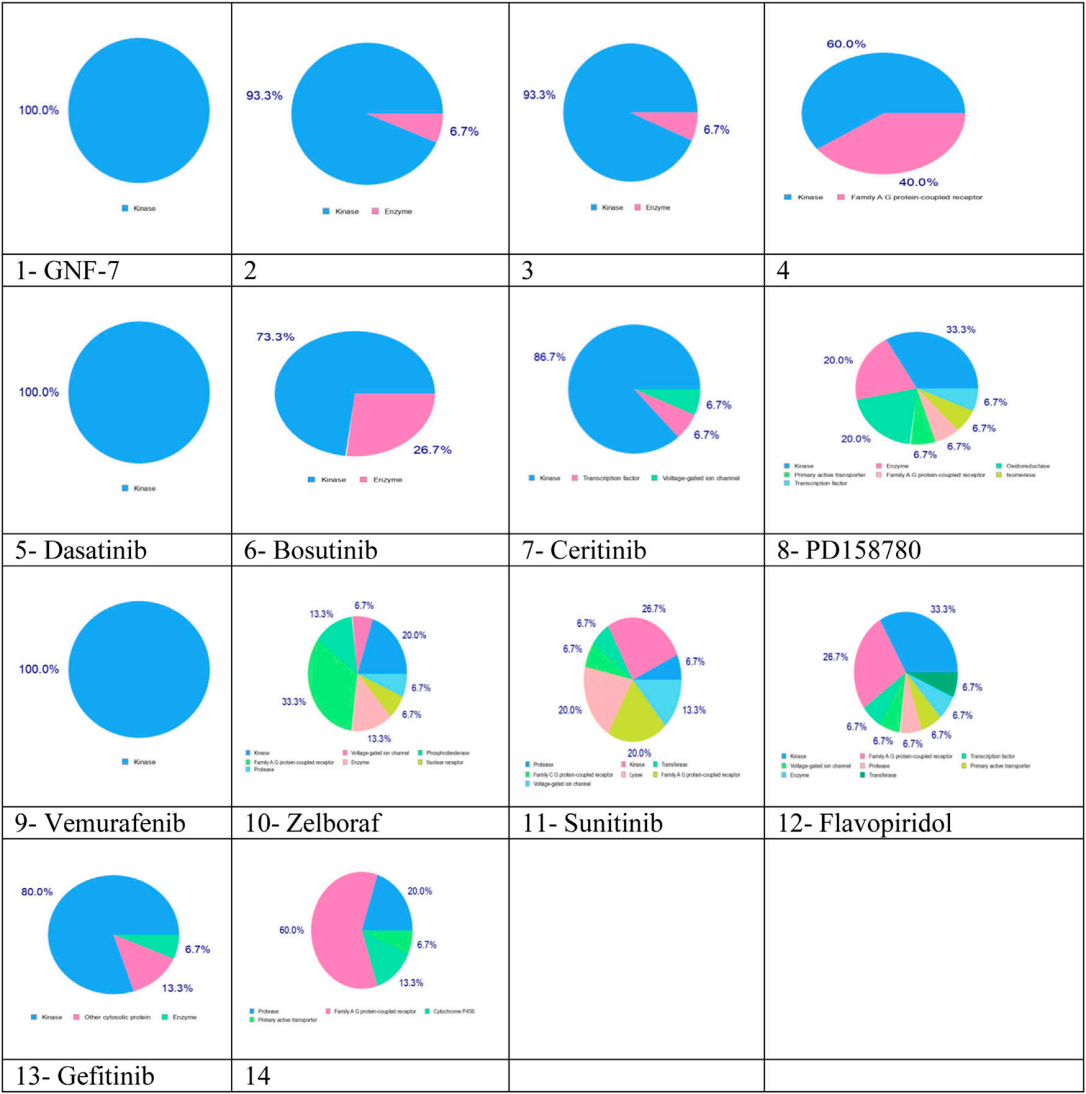
Predicted biological targets for 14 multikinase inhibitors using the SwissTargetPrediction online tool. Blue color: kinase, pink: family AG protein-coupled receptor, light green: voltage-gated ion channel, light pink: protease, green: transferase, light blue: transcription factor, and yellow: primary active transporter.

SwissTargetPrediction results indicated that these complexes can be used as multikinase inhibitors. Complexes 1–13 have kinase as the main potential target (blue color), while complexes 10, 11, 12, and 14 have the potential to target protease, transferase, enzyme, family A G protein-coupled receptor, and voltage-gated ion channel, as shown in Figure 4. The multitargeting activities of these inhibitors are validated by the experimental results (Wang Z.-Z. et al., 2021).

The docking of ACK1–inhibitors along with their binding energies is given in Supplementary Figure 2. It has been observed that drug-like and lead-like criteria for physiochemical properties were only applied to ligands whose receptor BE < −6 kcal/mol for the best docking pose. All the studied complexes have the best binding energy poses (BE < −6 kcal/mol), which reflect the importance of these non-receptors as potential druggable targets for target therapies. The binding of inhibitors is mostly toward the best binding pocket of ACK1, as predicted in Figure 3C.

Protein–Ligand Interaction Profiler (PLIP) predicted hydrophobic interactions (HYs) and hydrogen bonds (HB) in all ACK1–inhibitors, which enhance the binding affinity and biological activity of complex molecules and help in stabilizing the biochemical environments. Other interactions such as salt bridge (SB) was observed in ACK1-(3–6, 10, 13) complexes. SB and π–π stacking (perpendicular) interactions were observed in (ACK1-1, 9) complexes. π–π stacking (parallel) and no SB were seen in ACK1–9 complexes. ACK1–12 complexes have cation–π and SB interactions, while only cation–π interactions are seen in ACK1–10 complexes, as shown in Supplementary Figure 3. The π–π stacking is essential for the favorable electron correlation, whereas cation–π contacts produce further electrostatic contributions (Brylinski, 2018). These noncovalent interactions promote high-affinity binding of inhibitors to their targets which is important for the success of rational drug discovery.

## Conclusion

DFT results showed that the studied 14 inhibitors are highly stable. Most of the inhibitors are non-mutagenic, non-tumorigenic, non-irritant, and without effects on reproduction, and these inhibitors have good drug score values. Since cancer is a heterogeneous disease and variations have been observed in toxicity and ADMET results, it is recommended to use these inhibitors as combination drugs instead of single-drug treatment. ACK1–multikinase inhibitors show better binding affinities. They can be used as potential drugs for treating cancers and other diseases. Due to the multitarget activities of these inhibitors, we hope that these studies will also provide a reference and possibility for the study and treatment of other diseases in the future (Rahman et al., 2021).

## Data Availability

The original contributions presented in the study are included in the article/Supplementary Material, further inquiries can be directed to the corresponding author.

## References

[B1] Attwood M. M. Fabbro D. Sokolov A. V. Knapp S. Schiöth H. B. (2021). Trends in Kinase Drug Discovery: Targets, Indications and Inhibitor Design. Nat. Rev. Drug Discov. 20, 839–861. 10.1038/s41573-021-00252-y 34354255

[B3] Ayers P. W. Yang W. (2003). “Density Functional Theory,” in Computational Medicinal Chemistry for Drug Discovery. Editors Bultinck P. de Winter H. Langenaeker W. Tollenaere J. P. (New York: Dekker), 1–571.

[B4] Bollag G. Hirth P. Tsai J. Zhang J. Ibrahim P. N. Cho H. (2010). Clinical Efficacy of a RAF Inhibitor Needs Broad Target Blockade in BRAF-Mutant Melanoma. Nature 467, 596–599. 10.1038/nature09454 20823850 PMC2948082

[B5] Brylinski M. (2018). Aromatic Interactions at the Ligand-Protein Interface: Implications for the Development of Docking Scoring Functions. Chem. Biol. Drug Des. 91 (2), 380–390. 10.1111/cbdd.13084 28816025 PMC5818208

[B6] Chattaraj P. K. Nath S. Maiti B. (2003). Reactivity Descriptors. Computational Medicinal Chemistry for Drug Discovery. New York: Taylor & Francis, 1–295.

[B7] Chattaraj P. K. Poddar A. Maiti B. (2002). “Chemical Reactivity and Dynamics within a Density-Based Quantum Mechanical Framework,” in Reviews in Modern Quantum Chemistry: A Celebration of the Contributions of Robert Parr. Editor Sen K. D. (Singapore: World Scientific), 1–871. 10.1142/9789812775702_0030

[B8] Chen M.-L. Amidon G. L. Benet L. Z. Lennernas H. Yu L. X. (2011). The BCS, BDDCS, and Regulatory Guidances. Pharm. Res. 28 (7), 1774–1778. 10.1007/s11095-011-0438-1 21491148

[B9] Cho H. Shin I. Ju E. Choi S. Hur W. Kim H. (2018). First Sar Study for Overriding NRAS Mutant Driven Acute Myeloid Leukemia. J. Med. Chem. 61, 8353–8373. 10.1021/acs.jmedchem.8b00882 30153003

[B10] Choi H. G. Ren P. Adrian F. Sun F. Lee H. S. Wang X. (2010). A Type-II Kinase Inhibitor Capable of Inhibiting the T315I "Gatekeeper" Mutant of Bcr-Abl. J. Med. Chem. 53, 5439–5448. 10.1021/jm901808w 20604564 PMC4134510

[B11] Daina A. Michielin O. Zoete V. (2019). SwissTargetPrediction: Updated Data and New Features for Efficient Prediction of Protein Targets of Small Molecules. Nucleic Acids Res. 47, W357. 10.1093/nar/gkz382 31106366 PMC6602486

[B12] Daina A. Zoete V. (2016). A BOILED-Egg to Predict Gastrointestinal Absorption and Brain Penetration of Small Molecules. CHEMMEDCHEM 11 (11), 1117–1121. 10.1002/cmdc.201600182 27218427 PMC5089604

[B13] Dennington R. Keith T. A. Millam J. M. (2003). GaussView, Version 6. Shawnee Mission, KS: Semichem Inc.

[B14] Flores-Holguín N. Frau J. Glossman-Mitnik D. (2021) In Density Functional Theory; De Lazaro S. R. Da Silveira Lacerda L. H. Pontes Ribeiro R. A., Eds.; IntechOpen: London, UK,; Chapter 3, pp 57–67. ISBN 978-1-78985-167-0, eISBN 978-1-78985-168-7.

[B15] Flores-Holguín N. Frau J. Glossman-Mitnik D. (2019a). Chemical-Reactivity Properties, Drug Likeness, and Bioactivity Scores of Seragamides A–F Anticancer Marine Peptides: Conceptual Density Functional Theory Viewpoint. Computation 7, 52. 10.3390/computation7030052

[B16] Flores-Holguín N. Frau J. Glossman-Mitnik D. (2019b). Computational Prediction of Bioactivity Scores and Chemical Reactivity Properties of the Parasin I Therapeutic Peptide of Marine Origin through the Calculation of Global and Local Conceptual DFT Descriptors F. Theor. Chem. Acc. 138, 4158. 10.3390/molecules25184158

[B17] Flores-Holguín N. Frau J. Glossman-Mitnik D. (2020a). A Fast and Simple Evaluation of the Chemical Reactivity Properties of the Pristinamycin Family of Antimicrobial Peptides. Chem. Phys. Lett. 739, 137021. 10.1016/j.cplett.2019.137021

[B18] Flores-Holguín N. Frau J. Glossman-Mitnik D. (2020b). Conceptual DFT-Based Computational Peptidology of Marine Natural Compounds. Discodermins A–h. Mol. 25, 4158. 10.3390/molecules25184158 PMC7570683 32932850

[B19] Flores-Holguín N. Frau J. Glossman-Mitnik D. (2020c). Virtual Screening of Marine Natural Compounds by Means of Chemoinformatics and CDFT-Based Computational Peptidology. Mar. Drugs 18, 478. 10.3390/md18090478 PMC7551818 32962305

[B20] Fox M. Crafter C. Owen D. (2019). The Non-receptor Tyrosine Kinase ACK: Regulatory Mechanisms, Signalling Pathways and Opportunities for Attacking Cancer. Biochem. Soc. Trans. 47, 1715–1731. 10.1042/bst20190176 31845724

[B21] Frau J. Flores-Holguín N. Glossman-Mitnik D. (2018). Chemical Reactivity Properties, pKa Values, AGEs Inhibitor Abilities and Bioactivity Scores of the Mirabamides A-H Peptides of Marine Origin Studied by Means of Conceptual DFT. Mar. Drugs 16, 302–319. 10.3390/md16090302 PMC6163382 30154377

[B22] Frau J. Flores-Holguín N. Glossman-Mitnik D. (2019). Chemical Reactivity Theory and Empirical Bioactivity Scores as Computational Peptidology Alternative Tools for the Study of Two Anticancer Peptides of Marine Origin. Molecules 24, 1115. 10.3390/molecules24061115 PMC6470772 30901820

[B23] Frisch M. J. Trucks G. W. Schlegel H. B. Scuseria G. E. Robb M. A. Cheeseman J. R. (2016). Gaussian 16 Software Suite. Wallingford CT: Gaussian, Inc.

[B2] Hanwell M. D. Curtis D. E. Lonie D. C. Vandermeersch T. Zurek E. Hutchison G. R. (2016). Avogadro: An Advanced Semantic Chemical Editor, Visualization, and Analysis Platform. J. Cheminform. 4, 17–34. Available at: http://www.jcheminf.com/content/4/1/17 . 10.1186/1758-2946-4-17 PMC3542060 22889332

[B24] Jendele L. Krivak R. Skoda P. Novotny M. Hoksza D. (2019). PrankWeb: a Web Server for Ligand Binding Site Prediction and Visualization. Nucleic Acids Res. 47, W345–W349. 10.1093/nar/gkz424 31114880 PMC6602436

[B25] Kaya S. Kaya C. (2015a). A Simple Method for the Calculation of Lattice Energies of Inorganic Ionic Crystals Based on the Chemical Hardness. Inorg. Chem. 54, 8207–8213. 10.1021/acs.inorgchem.5b00383 26305871

[B26] Kaya S. Kaya C. (2015b). A New Equation for Calculation of Chemical Hardness of Groups and Molecules. Mol. Phys. 113, 1311–1319. 10.1080/00268976.2014.991771

[B27] Kohn W. Becke A. D. Parr R. G. (1996). Density Functional Theory of Electronic Structure. J. Phys. Chem. 100, 12974–12980. 10.1021/jp960669l

[B28] Ku M. S. (2008). Use of the Biopharmaceutical Classification System in Early Drug Development. AAPS J. 10 (1), 208–212. 10.1208/s12248-008-9020-0 18446521 PMC2751465

[B29] Kuriata A. Gierut A. M. Oleniecki T. Ciemny M. P. Kolinski A. Kurcinski M. (2018). CABS-flex 2.0: a Web Server for Fast Simulations of Flexibility of Protein Structures. Nucleic Acids Res. 46, W338–W343. 10.1093/nar/gky356 29762700 PMC6031000

[B30] La Torre A. del Mar Masdeu M. Cotrufo T. Moubarak R. S. del Río J. A. Comella J. X. (2013). A Role for the Tyrosine Kinase ACK1 in Neurotrophin Signaling and Neuronal Extension and Branching. Cell Death Dis 4, e602. 10.1038/cddis.2013.99 23598414 PMC3668633

[B31] Lin Q. Wang J. Childress C. Yang W. (2012). The Activation Mechanism of ACK1 (Activated Cdc42-Associated Tyrosine Kinase 1). Biochem. J. 445, 255–264. 10.1042/BJ20111575 22553920

[B32] Lipinski C. A. Lombardo F. Dominy B. W. Feeney P. J. (1997). Experimental and Computational Approaches to Estimate Solubility and Permeability in Drug Discovery and Development Settings. Adv. Drug Deliv. Rev. 23, 3–25. 10.1016/s0169-409x(96)00423-1 11259830

[B33] Liu Y. Karaca M. Zhang Z. Gioeli D. Earp H. S. Whang Y. E. (2010). Dasatinib Inhibits Site-specific Tyrosine Phosphorylation of Androgen Receptor by Ack1 and Src Kinases. Oncogene 29, 3208–3216. [PubMed: 20383201]. 10.1038/onc.2010.103 20383201 PMC2880659

[B34] Lombardo L. J. Lee F. Y. Chen P. Norris D. Barrish J. C. Behnia K. (2004). Discovery of N-(2-chloro-6-methyl- Phenyl)-2-(6-(4-(2-Hydroxyethyl)- Piperazin-1-Yl)-2-Methylpyrimidin-4- Ylamino)thiazole-5-Carboxamide (BMS-354825), a Dual Src/Abl Kinase Inhibitor with Potent Antitumor Activity in Preclinical Assays. J. Med. Chem. 47, 6658–6661. 10.1021/jm049486a 15615512

[B35] Mahajan N. P. Whang Y. E. Mohler J. L. Earp H. S. (2005). Activated Tyrosine Kinase Ack1 Promotes Prostate Tumorigenesis: Role of Ack1 in Polyubiquitination of Tumor Suppressor Wwox. Cancer Res. 65, 10514–10523. 10.1158/0008-5472.can-05-1127 16288044

[B36] Mahajan K. Coppola D. Challa S. Fang B. Chen Y. A. Zhu W. (2010). Ack1 Mediated AKT/PKB Tyrosine 176 Phosphorylation Regulates its Activation. PLoS One 5, e9646. 10.1371/journal.pone.0009646 20333297 PMC2841635

[B37] Mahajan K. Coppola D. Rawal B. Chen Y. A. Lawrence H. R. Engelman R. W. (2012). Ack1-mediated Androgen Receptor Phosphorylation Modulates Radiation Resistance in Castration-Resistant Prostate Cancer. J. Biol. Chem. 287, 22112–22122. 10.1074/jbc.M112.357384 22566699 PMC3381169

[B38] Mahajan K. Lawrence H. R. Lawrence N. J. Mahajan N. P. (2014). ACK1 Tyrosine Kinase Interacts with Histone Demethylase KDM3A to Regulate the Mammary Tumor Oncogene HOXA1. J. Biol. Chem. 289, 28179–28191. 10.1074/jbc.m114.584425 25148682 PMC4192474

[B39] Mahajan K. Mahajan N. P. (2010). Shepherding AKT and Androgen Receptor by Ack1 Tyrosine Kinase. J. Cel. Physiol. 224, 327–333. 10.1002/jcp.22162 PMC3953130 20432460

[B40] Mahajan K. Mahajan N. P. (2013). ACK1 Tyrosine Kinase: Targeted Inhibition to Block Cancer Cell Proliferation. Cancer Lett. 338, 185–192. 10.1016/j.canlet.2013.04.004 23597703 PMC3750075

[B41] Mahajan K. Mahajan N. P. (2015). ACK1/TNK2 Tyrosine Kinase: Molecular Signaling and Evolving Role in Cancers. Oncogene 34, 4162–4167. 10.1038/onc.2014.350 25347744 PMC4411206

[B42] Manser E. Leung T. Salihuddin H. Tan L. Lim L. (1993). A Non-receptor Tyrosine Kinase that Inhibits the GTPase Activity of P21cdc42. Nature 363, 364–367. 10.1038/363364a0 8497321

[B43] Marenich A. V. Cramer C. J. Truhlar D. G. (2009). Universal Solvation Model Based on Solute Electron Density and on a Continuum Model of the Solvent Defined by the Bulk Dielectric Constant and Atomic Surface Tensions. J. Phys. Chem. B 113, 6378–6396. 10.1021/jp810292n 19366259

[B44] Maxson J. E. Abel M. L. Wang J. Deng X. Reckel S. Luty S. B. (2016). Identification and Characterization of Tyrosine Kinase Nonreceptor 2 Mutations in Leukemia through Integration of Kinase Inhibitor Screening and Genomic Analysis. Cancer Res. 76, 127–138. 10.1158/0008-5472.can-15-0817 26677978 PMC4703549

[B45] Morris G. M. Huey R. Lindstrom W. Sanner M. F. Belew R. K. Goodsell D. S. (2009). AutoDock4 and AutoDockTools4: Automated Docking with Selective Receptor Flexibility. J. Comput. Chem. 30 (16), 2785–2791. 10.1002/jcc.21256 19399780 PMC2760638

[B46] Muegge I. Heald S. L. Brittelli D. (2001). Simple Selection Criteria for Drug-like Chemical Matter. J. Med. Chem. 44 (12), 1841–1846. 10.1021/jm015507e 11384230

[B47] Nur-E-Kamal A. Zhang A. Keenan S. M. Wang X. I. Seraj J. Satoh T. (2005). Requirement of Activated Cdc42-Associated Kinase for Survival of V-Ras-Transformed Mammalian Cells. Mol. Cancer Res. 3, 297–305. 10.1158/1541-7786.mcr-04-0152 15886301

[B48] O'boyle N. M. Tenderholt A. L. Langner K. M. (2008). Cclib: A Library for Package-independent Computational Chemistry Algorithms. J. Comput. Chem. 29, 839–845. 10.1002/jcc.20823 17849392

[B49] Osiris Property Explorer (2021). Available from: www.organicchemistry.org/prog/peo/ (Accessed, 08/12/2021)

[B50] Parr R. G. Szentpály L. v. Liu S. (1999). Electrophilicity Index. J. Am. Chem. Soc. 121, 1922–1924. 10.1021/ja983494x

[B51] Parr R. G. Yang W. (1989). Density Functional Theory of Atoms and Molecules. Oxford: Oxford University Press, 1–333.

[B52] Parr R. G. Yang W. (1995). Density-functional Theory of the Electronic Structure of Molecules. Annu. Rev. Phys. Chem. 46, 701–728. 10.1146/annurev.pc.46.100195.003413 24341393

[B53] Phatak S. S. Zhang S. (2013). A Novel Multi-Modal Drug Repurposing Approach for Identification of Potent ACK1 Inhibitors. Pac. Symp. Biocomput, 29–40. 10.1142/9789814447973_0004 23424109 PMC3864554

[B54] Pitsillou E. Liang J. Ververis K. Lim K. W. Hung A. Karagiannis T. C. (2020). Identification of Small Molecule Inhibitors of the Deubiquitinating Activity of the SARS-CoV-2 Papain-like Protease: In Silico Molecular Docking Studies and *In Vitro* Enzymatic Activity Assay. Front. Chem. 8, 623971. 10.3389/fchem.2020.623971 33364229 PMC7753156

[B55] Prieto-Echagüe V. Gucwa A. Craddock B. P. Brown D. A. Miller W. T. (2010). Cancer-associated Mutations Activate the Nonreceptor Tyrosine Kinase Ack1. J. Biol. Chem. 285, 10605–10615. 10.1074/jbc.m109.060459 20110370 PMC2856269

[B56] Rahman M. Talukder A. Akter R. (2021). Computational Designing and Prediction of ADMET Properties of Four Novel Imidazole‐based Drug Candidates Inhibiting Heme Oxygenase‐1 Causing Cancers. Mol. Inf. 40, 2060033–2060047. 10.1002/minf.202060033 34241977

[B57] Salentin S. Schreiber S. Haupt V. J. Adasme M. F. Schroeder M. (2015). PLIP: Fully Automated Protein–Ligand Interaction Profiler. Nucleic Acids Res. 43, W443–W447. 10.1093/nar/gkv315 25873628 PMC4489249

[B58] Sarkar U. Chattaraj P. K. (2021). Reactivity Dynamics. J. Phys. Chem. A. 125, 2051–2060. 10.1021/acs.jpca.0c10788 33566617

[B59] Simulations Plus Inc (2022). Available from: https://www.simulations-plus.com/software/admetpredictor/2022 .

[B60] Srivastava R. (2021a). Theoretical Studies on the Molecular Properties, Toxicity, and Biological Efficacy of 21 New Chemical Entities. ACS Omega 6 (38), 24891–24901. 10.1021/acsomega.1c03736 34604670 PMC8482469

[B61] Srivastava R. (2021b). Chemical Reactivity Theory (CRT) Study of Small Drug-like Biologically Active Molecules. J. Biomol. Struct. Dyn. 39 (3), 943–952. 10.1080/07391102.2020.1725642 32008483

[B62] Stansfield L. Hughes T. E. Walsh-Chocolaad T. L. (2013). Bosutinib. Ann. Pharmacother. 47, 1703–1711. 10.1177/1060028013503124 24396109

[B63] Verduzco D. Kuenzi B. M. Kinose F. Sondak V. K. Eroglu Z. Rix U. (2018). Ceritinib Enhances the Efficacy of Trametinib in BRAF/NRAS-wild-type Melanoma Cell Lines. Mol. Cancer Ther. 17, 73–83. 10.1158/1535-7163.mct-17-0196 29133622 PMC5752595

[B64] von Szentpály L. (2017). Hardness Maximization or Equalization? New Insights and Quantitative Relations between Hardness Increase and Bond Dissociation Energy. J. Mol. Model. 23, 217. 10.1007/s00894-017-3383-z 28669126

[B65] von Szentpály L. (2018a). Eliminating Symmetry Problems in Electronegativity Equalization and Correcting Self-Interaction Errors in Conceptual DFT. J. Comput. Chem. 39, 1949–1969. 10.1002/jcc.25356 30144124

[B66] Von Szentpaly L. (2018b). Multiply Charged Anions, Maximum Chargé Acceptance, and Higher Electron Affinities of Molecules, Superatoms, and Clusters. Acta Phys.-Chim. Sin. 34, 675–682. 10.3866/PKU.WHXB201801021

[B67] Von Szentpaly L. (2000). Modeling the Charge Dependence of Total ´ Energy and its Relevance to Electrophilicity. Int. J. Quan. Chem. 76, 222–234. 10.1002/(SICI)1097-461X(2000)76:2<222::AID-QUA11

[B68] Von Szentpály L. (2020). Theorems and Rules Connecting Bond Energy and Bond Order with Electronegativity Equalization and Hardness Maximization. Theor. Chem. Acc. 139, 54. 10.1007/s00214-020-2569-0

[B69] von Szentpály L. Kaya S. Karakuş N. (2020). Why and when Is Electrophilicity Minimized? New Theorems and Guiding Rules. J. Phys. Chem. A. 124 (51), 10897–10908. 10.1021/acs.jpca.0c08196 33301330

[B70] Wang A. Pei J. Shuai W. Lin C. Feng L. Wang Y. (2021a). Small Molecules Targeting Activated Cdc42-Associated Kinase 1 (ACK1/TNK2) for the Treatment of Cancers. J. Med. Chem. 64 (22), 16328–16348. 10.1021/acs.jmedchem.1c01030 34735773

[B71] Wang Z.-Z. Shi X.-X. Huang G.-Y. Hao G.-F. Yang G.-F. (2021b). Fragment-based Drug Design Facilitates Selective Kinase Inhibitor Discovery. Trends Pharmacol. Sci. 42, 551–565. 10.1016/j.tips.2021.04.001 33958239

[B72] Zhao X. Lv C. Chen S. Zhi F. (2018). A Role for the Non-receptor Tyrosine Kinase ACK1 in TNF-Alpha-Mediated Apoptosis and Proliferation in Human Intestinal Epithelial Caco-2 Cells. Cell Biol. Int. 42, 1097–1105. 10.1002/cbin.10875 28921811

[B73] Zhao Y. Truhlar D. G. (2008). The M06 Suite of Density Functionals for Main Group Thermochemistry, Thermochemical Kinetics, Noncovalent Interactions, Excited States, and Transition Elements: Two New Functionals and Systematic Testing of Four M06-Class Functionals and 12 Other Functionals. Theor. Chem. Account. 120, 215–241. 10.1007/s00214-007-0310-x

