# Supplementary material for: Chemical Reactivity and Optical and Pharmacokinetics Studies of 14 Multikinase Inhibitors and Their Docking Interactions Toward ACK1 for Precision Oncology
Source: Front Chem. 2022 Apr 14;10:843642. doi: 10.3389/fchem.2022.843642 (PMC9050413; doi:10.3389/fchem.2022.843642)
Supplement: Supplementary file 1 [file DataSheet1.PDF]

Supplementary Information

Title: Chemical Reactivity, Optical and Pharmacokinetics Studies of 14 Multikinase Inhibitors and its Docking Interactions towards ACK1 for Precision Oncology

Submitted by: Ruby Srivastava*

Principal Investigator, Bioinformatics

CSIR-CCMB, India.

Formulas Used

The ionization potential (IP) and electron affinity (EA) values were calculated using the finite difference approximation, and are given by:

IP = E(N-1) – E(N)

EA= E(N) – E(N+1)

Global parameters: The molecular descriptors such as the energy of the highest occupied molecular orbital (E_{HOMO}), the energy of the lowest unoccupied molecular orbital (E_{LUMO}), the energy gap

($\Delta E = E_{LUMO} - E_{HOMO}$).

The reactivity parameters were then calculated using the conceptual framework of DFT. So, the chemical potential μ_p is defined as:

$$\mu_p = (\partial E / \partial N)_{v(r)} = -\chi$$

where μ_p is the chemical potential, E is the total energy, N is the number of electrons, v(r) is the external potential of the system and χ is the global electronegativity. The global hardness is given by:

$$\eta = (\partial^2 E / \partial N^2)_{(r)}$$

Using the finite difference approximation and the Koopmans theorem, the global electronegativity and the global hardness are given by:

$$\chi = (I+A)/2 \approx -(E_{HOMO}+E_{LUMO})/2$$

$\eta = (I-A)/2 \approx (E_{LUMO}-E_{HOMO})/2$ respectively.

The global softness which is the reciprocal of the global hardness can be obtained by:

$$S = 1/\eta$$

The electrophilicity index (ω) is defined as:

$$\omega = \mu_p^2/2\eta.$$

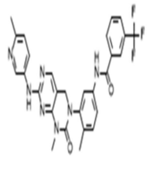

1

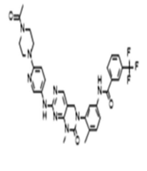

2

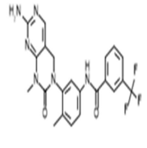

3

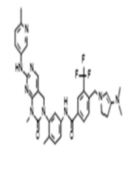

4

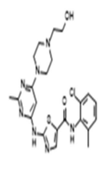

5

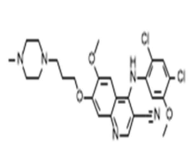

6

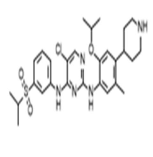

7

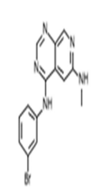

8

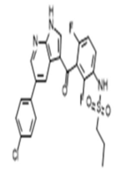

9

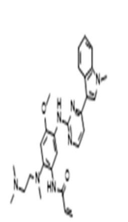

10

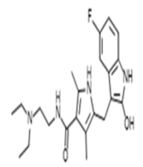

11

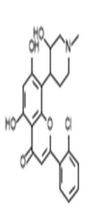

12

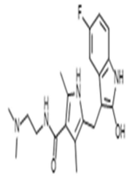

13

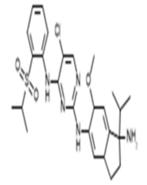

14

Supplementary Figure 1. The chemical structures of 14 Multikinase Inhibitors.

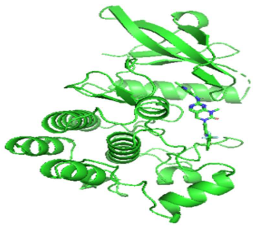	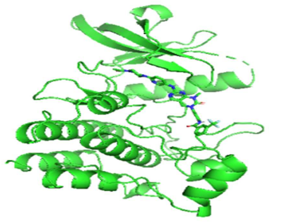	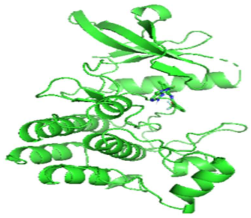	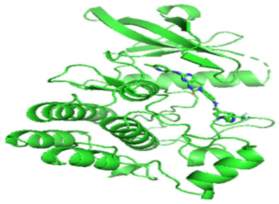
ACK1-1 BE (-7.78)	ACK1-2 BE (-9.86)	ACK1-3 BE (-7.09)	ACK1-4 BE (-8.16)
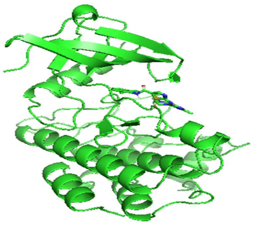	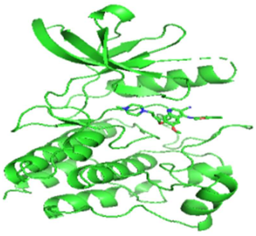	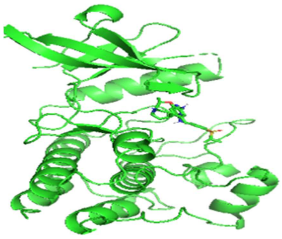	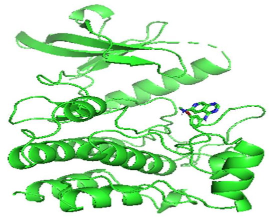
ACK1-5 BE (-7.66)	ACK1-6 BE (-6.96)	ACK1-7 BE (-7.52)	ACK1-8 BE (-6.23)
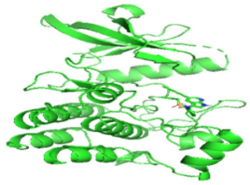	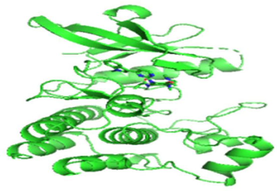	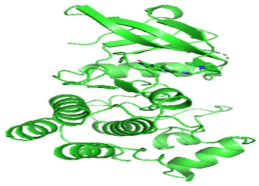	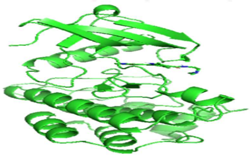
ACK1-9 BE (-7.12)	ACK1-10 BE (-7.68)	ACK1-11 BE (-8.15)	ACK1-12 BE (-6.18)
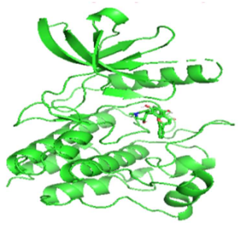	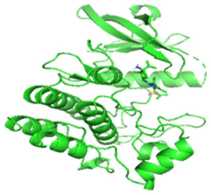		
ACK1-13 BE (-8.29)	ACK1-14 BE (-7.98)		

Supplementary Figure 2. Docking Interactions and Binding energies (BE) (kcal/mol) of ACK1-Multikinase Inhibitors using Autodock 4.2.6 software tools.

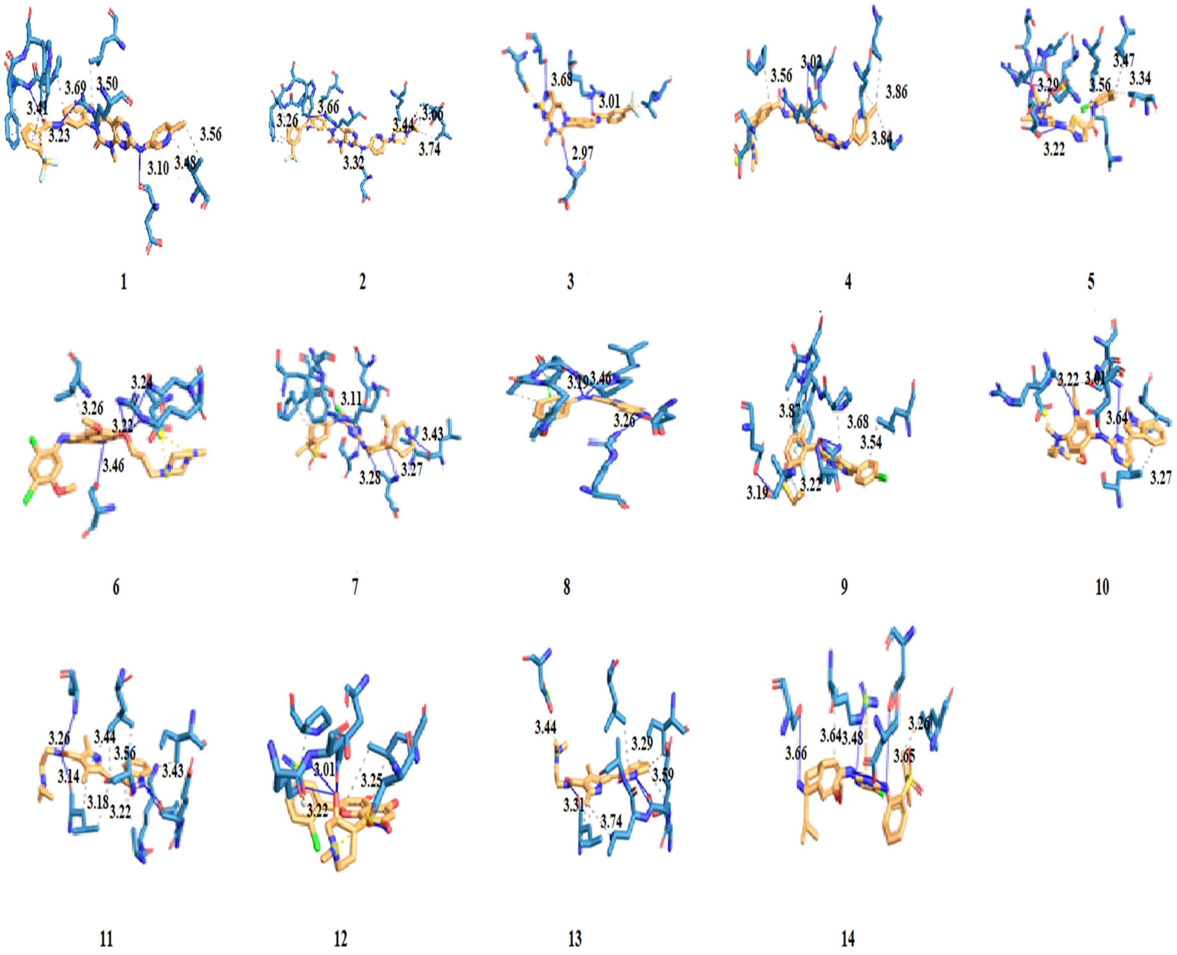

Supplementary Figure 3. Protein Ligand Interaction for ACK1-Multikinase inhibitors from Protein Ligand Interaction Profiler (PLIP) online tool. Color codes are Blue-Protein, Light orange-Ligand, Light blue-Water, Dotted blue line-Hydrophobic Interaction, Smooth blue line-Hydrogen Bond. The bond distances are given in angstrom (Å) unit.

Structure	Identifier	*Risks*	*CYP Substr*	*CYP CLint*	*UGT Substr*
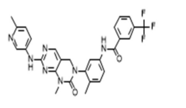	1 (GNF-7)	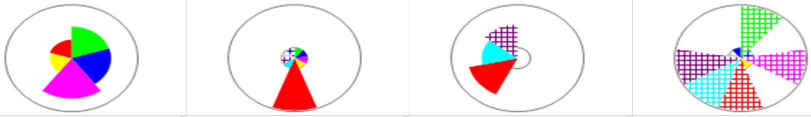			
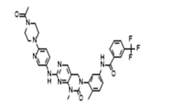	2	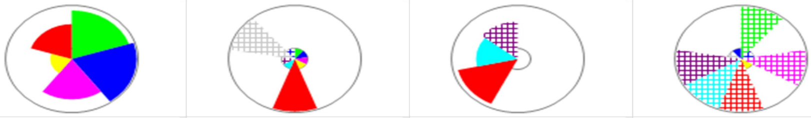			
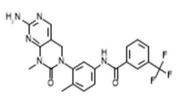	3	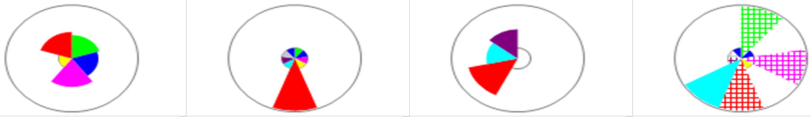			
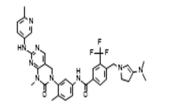	4	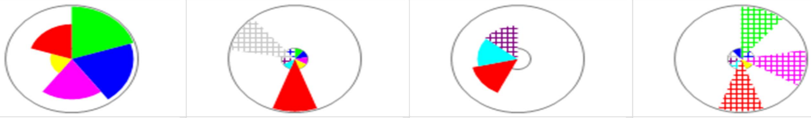			
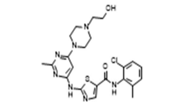	5 (Dasatinib)	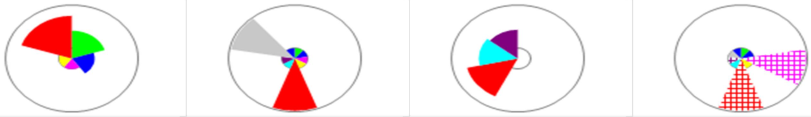			
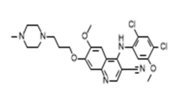	6 (Bosutinib)	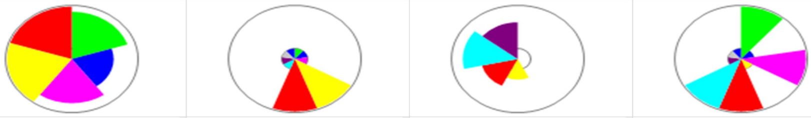			
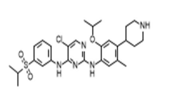	7 (Ceritinib)	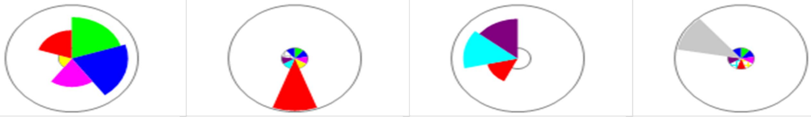			
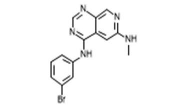	8 (PD158780	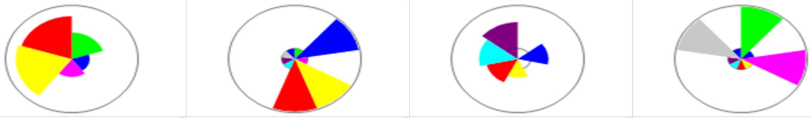			
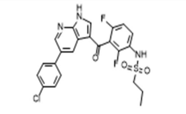	9 (Vemurafen	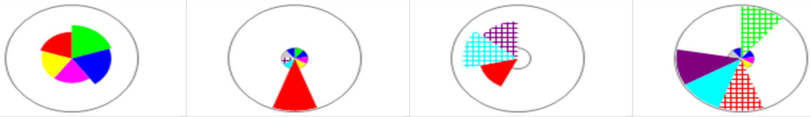			
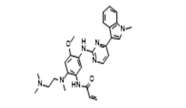	10 (ADZ9291	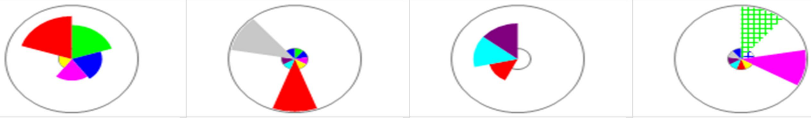			
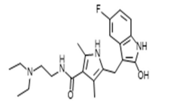	11 (Sunitinib)	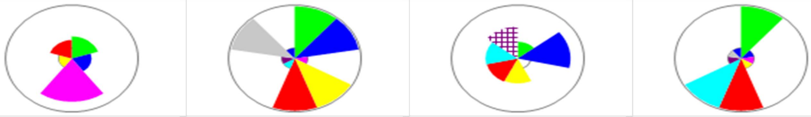			
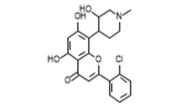	12 (Flavopiric	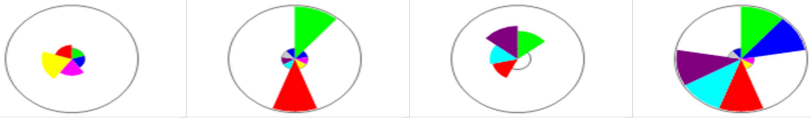			
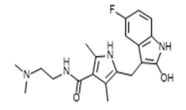	13 (Gefitinib)	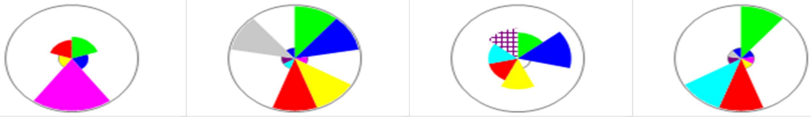			
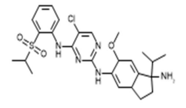	14	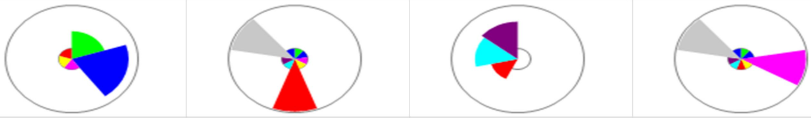			

Supplementary Table 4: The chemical structures, names and graphical representation of risk and CYP substrate classification (CYP Sus, CYP CLinh) and UGT subs) for 14 Multikinase Inhibitors as predicted by Simulationplus ADMET Predictor Software. Short wedges indicate that the compound is not a substrate and wedge extending out to the edge indicate that inhibitor is a substrate of the corresponding enzyme.

Identifier	Structure	*TRNS Substr*	*TRNS Inh*
1 (GNF-7)	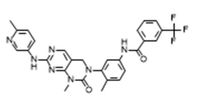	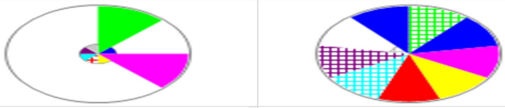	
2	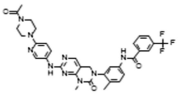	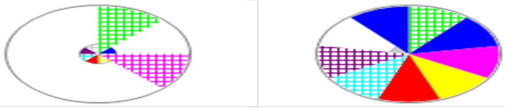	
3	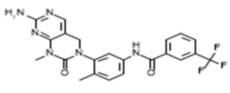	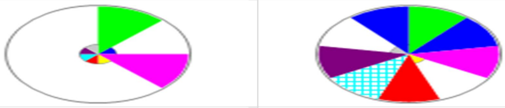	
4	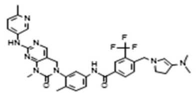	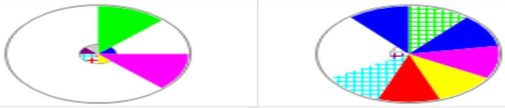	
5 (Dasatinib)	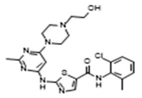	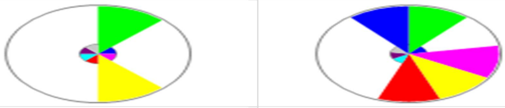	
6 (Bosutinib)	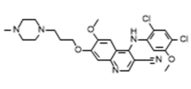	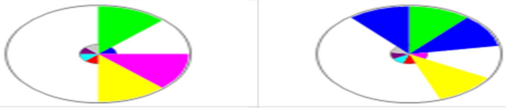	
7 (Ceritinib)	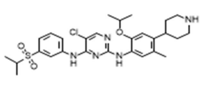	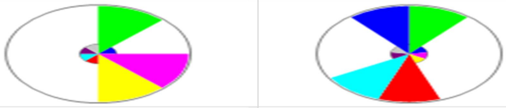	
8 (PD158780)	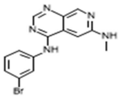	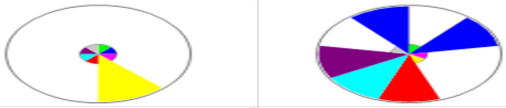	
9 (Vemurafenib)	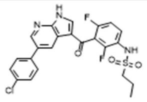	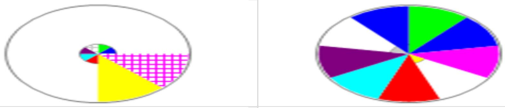	
10 (ADZ9291)	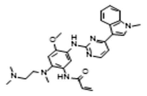	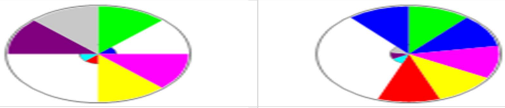	
11 (Sunitinib)	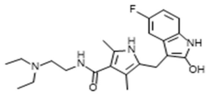	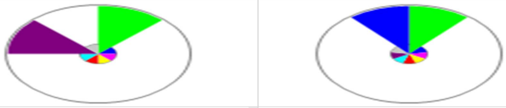	
12 (Flavopiridol)	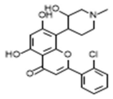	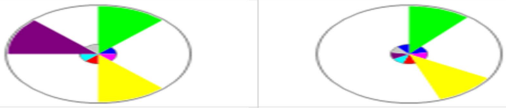	
13 (Gefitinib)	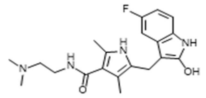	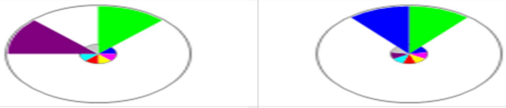	
14	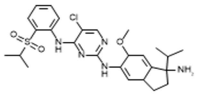	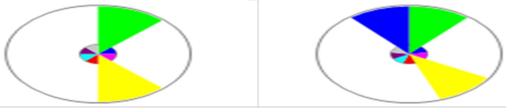	

Supplementary Table 5: The chemical structures, names and graphical representation of transporters substrate (TRNS(subs)) and transporters inhibitor (TRNS(inh)) for 14 Multikinase Inhibitors as predicted by Simulationplus ADMET Predictor 10.2. Short wedges

indicate that the compound is not a substrate and wedge extending out to the edge indicate that inhibitor is a substrate of the corresponding Transporter.

The full form of all toxicity parameters are given in ADMET Predictor manual.

Optimized Coordinates of studied 14 Multikinase Inhibitors

Complex 1

C	-9.48211000	-2.48648500	-0.62157900
C	-10.07332300	-1.47159600	0.14077400
C	-9.28090500	-0.45510800	0.65582900
C	-7.90105200	-0.45743500	0.40586000
C	-7.39453000	-1.51606600	-0.37217500
N	-8.16223600	-2.49163200	-0.86400300
H	-9.72505900	0.34036600	1.25030800
H	-11.14255900	-1.47772600	0.32895100
H	-6.33728200	-1.56048100	-0.59556000
N	-7.14248800	0.59122800	0.94109700
H	-7.65776300	1.29975400	1.44574100
C	1.37743800	0.93192400	-0.02926200
C	0.26254200	1.69908500	-0.36410200
C	0.40946300	2.97354900	-0.94008900
C	1.71610200	3.43380800	-1.14349800
C	2.84221500	2.68360700	-0.81803600
C	2.67280800	1.40678400	-0.26334800
H	1.22456000	-0.05761000	0.38994000
H	1.85894200	4.41406200	-1.59044600
H	3.83795600	3.05992400	-1.00036400
C	6.47285800	-2.29372500	1.80227300
C	5.58915700	-1.29012700	1.40476900
C	5.97562300	-0.34272100	0.44709100
C	7.26776700	-0.40051400	-0.08751200
C	8.14482200	-1.40818700	0.30601800
C	7.74988800	-2.36113500	1.25010000
H	6.16605200	-3.02066700	2.54747000
H	4.61136200	-1.23525600	1.87498700
H	7.56443900	0.34954700	-0.81098700
H	8.43797600	-3.14530100	1.54591000
C	9.54773600	-1.43782600	-0.24334100
F	9.61408700	-0.90945100	-1.48287500
F	10.39983800	-0.73217600	0.53521100

F	10.03056800	-2.69944800	-0.30959400
N	3.74314700	0.55481000	0.08563900
H	3.46865000	-0.37757300	0.35716600
C	5.09481600	0.78217100	-0.03249000
O	5.58052400	1.80649800	-0.49913600
C	-0.77530000	3.81929300	-1.33699900
H	-1.60845800	3.20285000	-1.68409800
H	-1.14760800	4.42651700	-0.50106800
H	-0.50150200	4.51253300	-2.13666300
C	-4.17708200	2.30488200	1.60665100
N	-5.47878900	2.01135200	1.57077200
C	-5.79716000	0.86786500	0.92863500
C	-3.65494300	0.36761600	0.36268800
H	-3.90645800	3.22151100	2.13046100
C	-3.19373600	1.51157600	1.03349400
N	-4.94775900	0.03399200	0.30852200
C	-1.44323700	-0.01267100	-0.62019800
N	-2.74181900	-0.45854200	-0.27385800
C	-1.71764200	1.74194900	1.12070600
N	-1.05563200	1.20053600	-0.07668600
H	-1.49241800	2.80933300	1.18526400
O	-0.74312800	-0.66214900	-1.38136000
C	-3.20709700	-1.70520000	-0.88937200
H	-3.79390900	-2.26770200	-0.16264500
H	-3.83244800	-1.50401800	-1.76423600
H	-2.32920300	-2.26770800	-1.19619000
H	-1.29177100	1.27315400	2.02448300
C	-10.28477100	-3.62079900	-1.20752600
H	-9.93622900	-4.58166700	-0.81428800
H	-11.35000900	-3.52105900	-0.98283800
H	-10.15993600	-3.65719600	-2.29457700

Complex 2

C	-7.55865800	-0.10699300	-0.44438700
C	-7.87529800	-1.37098600	-0.99077200
C	-6.85848500	-2.29163400	-1.17504600
C	-5.53771800	-1.98413800	-0.81209000
C	-5.32663400	-0.71811700	-0.24700000
N	-6.30111400	0.17809100	-0.07417100
H	-7.09131700	-3.26689300	-1.59705200
H	-8.88751600	-1.64555000	-1.25670600
H	-4.33485100	-0.42812600	0.07285300
N	-4.55457700	-2.96620600	-1.01386400
H	-4.88149400	-3.86346500	-1.34500400
C	3.74370900	-1.22030100	0.24033300
C	2.79688700	-2.11300700	0.74075700
C	3.17191000	-3.14485100	1.61922900
C	4.52950500	-3.23797100	1.94997000
C	5.48996800	-2.35616700	1.46310500
C	5.09086700	-1.32204500	0.60414200
H	3.41322400	-0.42065300	-0.41506000
H	4.84658200	-4.02426100	2.62990900
H	6.52785200	-2.44471300	1.74859600
C	8.13221000	2.52090300	-2.28062100
C	7.46892600	1.46535300	-1.65436300
C	7.97528300	0.91131400	-0.47107200
C	9.16914700	1.41203800	0.06142300

C	9.82377600	2.47153600	-0.56136600
C	9.30600800	3.03275300	-1.73326500
H	7.73462700	2.94097100	-3.19898600
H	6.57417600	1.05859600	-2.11715900
H	9.56468400	0.96193900	0.96414400
H	9.81975900	3.86150700	-2.20795600
C	11.12733400	2.98452500	-0.00612300
F	11.23096500	2.76874500	1.32125400
F	12.18760700	2.38174300	-0.59145100
F	11.26753800	4.31320700	-0.21716600
N	5.97514400	-0.36140900	0.06739900
H	5.52628200	0.38764900	-0.43846400
C	7.33047100	-0.23382800	0.26645700
O	7.99120200	-0.96934500	0.99111300
C	2.17007400	-4.11371700	2.19731400
H	1.19591600	-3.64050600	2.34436900
H	2.01394200	-4.98310800	1.54473500
H	2.51742400	-4.49716500	3.16035800
C	-1.26748900	-4.12936600	-1.11333100
N	-2.59891500	-4.11903800	-1.19699800
C	-3.19078700	-2.94584600	-0.88365200
C	-1.24380200	-1.88051400	-0.39716700
H	-0.77804900	-5.06833000	-1.37188800
C	-0.51071200	-3.02861200	-0.73563300
N	-2.57645100	-1.82496500	-0.46877200
C	0.76028100	-0.80770900	0.51855400
N	-0.56702000	-0.74811200	0.03157500
C	0.98383000	-2.95471100	-0.71713700
N	1.42663100	-2.00519700	0.31670300
H	1.41983000	-3.93208000	-0.49546800
O	1.25134900	0.14378900	1.10647100
C	-1.31831300	0.48294000	0.29291600
H	-1.96776000	0.69261400	-0.55712900
H	-1.93633100	0.38616400	1.19088000
H	-0.59847400	1.28427300	0.43851800
H	1.38265000	-2.64550600	-1.69872900
C	-10.67621100	1.94076200	-0.80568800
C	-9.92387600	0.64631100	-0.48354100
N	-8.50205400	0.91162500	-0.28633700
C	-8.22950700	1.95212100	0.70734100
C	-8.97968400	3.23991000	0.36519700
N	-10.40169100	2.97100800	0.18780800
H	-11.75275200	1.77075000	-0.81870600
H	-10.36973400	0.16903800	0.40608100
H	-7.15534200	2.13015700	0.72564300
H	-8.56755100	3.66376900	-0.56274200
H	-10.05706400	-0.03028400	-1.32975700
H	-10.36225700	2.29828200	-1.79715300
H	-8.82792200	3.96672300	1.16293800
H	-8.53693700	1.61353300	1.71200200
C	-11.44702300	3.64225000	0.78166700
O	-12.61597000	3.36877500	0.52818000
H	-12.03898300	5.15281800	2.14306400
H	-10.52841500	4.35260600	2.62985600
H	-10.51257300	5.54040300	1.31774700
C	-11.09914300	4.73934100	1.77935500

Complex 3

N	8.85535700	-1.53609700	-0.28350300
H	9.64088600	-1.04284400	-0.67636000
C	0.54983400	0.48320200	-0.01111400
C	1.77010100	1.12912000	0.18384700
C	1.82421700	2.51832300	0.39591900
C	0.60826600	3.21282400	0.38741700
C	-0.61989600	2.58679800	0.19664500
C	-0.65282400	1.19788600	0.00563600
H	0.54431000	-0.59383500	-0.14608600
H	0.62130300	4.28683700	0.55322900
H	-1.54306600	3.14716900	0.21010400
C	-4.95679800	-2.28885100	-1.29517100
C	-3.93070500	-1.36248600	-1.10649600
C	-4.18131600	-0.14911300	-0.45198100
C	-5.47992300	0.13394900	-0.01243200
C	-6.49995500	-0.79588100	-0.19582000
C	-6.24183700	-2.01298000	-0.83490400
H	-4.75375600	-3.22427600	-1.80670900
H	-2.94471400	-1.58288500	-1.50569100
H	-5.66854500	1.08429400	0.47245000
H	-7.04144800	-2.73343600	-0.96808200
C	-7.90492400	-0.47146600	0.24162400
F	-7.92650300	0.41907400	1.25438300
F	-8.62874600	0.05910600	-0.77084300
F	-8.56422400	-1.57794600	0.65522700
N	-1.84188100	0.45925600	-0.18001100
H	-1.71893100	-0.54239000	-0.18458200
C	-3.13933100	0.91411200	-0.21595900
O	-3.46114900	2.08692900	-0.06043700
C	3.12431100	3.24637200	0.63294900
H	3.85029500	2.61035000	1.14559800
H	3.58789900	3.57712200	-0.30601800
H	2.95893600	4.14135800	1.23876400
C	6.29434200	0.48320600	-1.56989400
N	7.52837300	0.00357300	-1.37612700
C	7.62434500	-0.97370700	-0.45622000
C	5.41772600	-0.95670500	0.08207300
H	6.19899500	1.26954600	-2.31889300
C	5.17554000	0.03211800	-0.88791000
N	6.62698200	-1.46858700	0.30483900
C	3.15387000	-0.69926000	0.98569500
N	4.35881000	-1.42510600	0.84896100
C	3.76346300	0.46660800	-1.12672700
N	2.99171400	0.37345800	0.12369700
H	3.72715900	1.50132800	-1.47669900
O	2.33613300	-1.00069000	1.84243500
C	4.59329100	-2.51690700	1.79764200
H	5.11895900	-3.32476400	1.28851500
H	5.20591800	-2.18257100	2.64043700
H	3.62552800	-2.84951100	2.16413600
H	3.28004300	-0.14806500	-1.90546200
H	8.99933600	-2.09389200	0.54255300

Complex 4

C	10.85594500	2.87654800	-1.83575800
C	11.44874300	2.71771000	-0.57722500
C	10.74713600	2.07220600	0.43157800

C	9.45603600	1.58766700	0.17723000
C	8.94106200	1.79091700	-1.11754100
N	9.62093100	2.41368900	-2.08338600
H	11.19382700	1.94176700	1.41487600
H	12.44903600	3.09573500	-0.38937100
H	7.95042900	1.43328200	-1.36297600
N	8.78776600	0.93833900	1.22265000
H	9.30557600	0.83423900	2.08472300
C	0.74382200	-1.90227300	0.33837000
C	2.00149500	-2.44538600	0.59653700
C	2.14653500	-3.80083500	0.94041900
C	0.97617200	-4.56544700	1.02117300
C	-0.28837800	-4.04372500	0.76525700
C	-0.40896500	-2.69295500	0.40707900
H	0.67393400	-0.85748100	0.05198300
H	1.05867100	-5.61757500	1.28084100
H	-1.17198200	-4.66221300	0.82169900
C	-5.00276200	0.55674700	-0.43012100
C	-3.92484900	-0.25848700	-0.08910500
C	-4.00578200	-1.64655700	-0.23784800
C	-5.20109500	-2.19903800	-0.71113900
C	-6.27776700	-1.38701200	-1.05593000
C	-6.19465000	0.01661700	-0.92203300
H	-4.93816800	1.63110000	-0.29998400
H	-3.03664700	0.20759200	0.32881900
H	-5.27044700	-3.27577700	-0.80048300
C	-7.53875900	-2.04206700	-1.56535800
F	-7.39300000	-3.36817800	-1.74794900
F	-8.57820600	-1.87363100	-0.70861900
F	-7.93171700	-1.51932800	-2.75439800
N	-1.63739600	-2.06621200	0.11077000
H	-1.55670000	-1.12321000	-0.23931000
C	-2.90332800	-2.60769100	0.11476100
O	-3.14386200	-3.78311900	0.36926300
C	3.49433700	-4.42426500	1.20820800
H	4.27650900	-3.96357600	0.59955200
H	3.79571000	-4.31836200	2.25896800
H	3.47272300	-5.49532700	0.99009100
C	6.06155600	-0.67044100	2.69072400
N	7.28389000	-0.15642100	2.53502000
C	7.53015300	0.39527400	1.32848800
C	5.47918200	-0.08704800	0.47876800
H	5.84735300	-1.11138500	3.66411600
C	5.08839800	-0.65476700	1.70215500
N	6.69023000	0.44340100	0.28305400
C	3.45212300	-0.92075600	-0.61635600
N	4.58268300	-0.06821700	-0.57740700
C	3.67711800	-1.12971500	1.85365600
N	3.16237200	-1.59621500	0.55687700
H	3.61909500	-1.95822200	2.56426300
O	2.81087100	-1.05861900	-1.64633200
C	4.95459400	0.60785000	-1.82393500
H	5.29437000	1.61885700	-1.59718400
H	5.76045700	0.07425300	-2.33647900
H	4.07425600	0.62959500	-2.46092200
H	3.02542700	-0.33013400	2.24597600
C	11.56363600	3.56728800	-2.97440100
H	10.97953000	4.42399500	-3.32609800

H	12.55531200	3.92060100	-2.67948300
H	11.67639900	2.88743800	-3.82544200
C	-7.33880300	0.93610300	-1.32842600
H	-7.33317200	1.02094500	-2.42171900
H	-8.30367800	0.47772000	-1.06209500
C	-7.47865500	2.46461100	0.59223900
C	-8.05859500	3.66822400	0.85368800
C	-8.15428900	4.44153600	-0.44774300
C	-7.99044400	3.31752200	-1.48935400
N	-7.21220700	2.28111300	-0.78671000
H	-7.24347000	1.67082700	1.28492800
H	-9.10002800	4.97934200	-0.56608900
H	-8.98335200	2.92497000	-1.76822200
N	-8.49460700	4.17106000	2.06761900
C	-8.26916800	5.59076400	2.32144300
C	-8.26364200	3.31843100	3.22151000
H	-7.21168900	5.81497900	2.53855700
H	-8.57577300	6.18371200	1.45749300
H	-8.87357800	5.90422000	3.17736400
H	-8.73727700	3.76088200	4.10140500
H	-8.71148500	2.33569700	3.04629100
H	-7.18723500	3.18069500	3.42256900
H	-5.55716500	3.32051700	1.61861500
H	-7.48087700	3.64043400	-2.40199300
H	-7.33995800	5.17494900	-0.52551800

Complex 5

C	-2.54127800	2.25242700	0.47382800
N	-1.43651300	1.51631200	0.27775200
C	-1.64886200	0.21904400	0.05894200
C	-2.91997500	-0.36099900	0.01056400
C	-4.01184200	0.50718300	0.19389900
N	-3.80173900	1.82113800	0.44458500
H	-3.03263700	-1.42794100	-0.12948900
C	-7.68900400	0.74142500	-0.09885800
C	-6.38815800	0.94116800	0.67145900
N	-5.32303900	0.09763100	0.12100900
C	-5.69127600	-1.30647700	-0.01596300
C	-7.02145000	-1.44851000	-0.75949000
N	-8.07252400	-0.67242600	-0.10789300
H	-8.47182200	1.32903300	0.38966500
H	-6.06011700	1.97746200	0.61901100
H	-4.92757000	-1.83476200	-0.58876100
H	-7.30733200	-2.50578900	-0.76009800
C	-10.52739700	-0.58881500	0.26215300
C	-9.38801800	-0.90522000	-0.70397800
H	-10.39827900	-1.19419000	1.17159400
H	-10.50071000	0.46962600	0.56381200
H	-9.46956100	-1.96505500	-0.96677400
H	-9.53722200	-0.33426100	-1.63828300
O	-11.73800300	-0.89678700	-0.41861300
H	-12.47344100	-0.70368200	0.17543800

H	-7.57296000	1.13294200	-1.12838700
H	-6.55437700	0.68356700	1.72909300
H	-5.77233400	-1.78484700	0.97302100
H	-6.87417100	-1.14132400	-1.81366800
C	-2.31922800	3.71625800	0.74734100
H	-1.69250700	3.83954000	1.63606900
H	-1.78006300	4.17543300	-0.08729000
H	-3.27061000	4.22821800	0.89377400
N	-0.54626100	-0.61606000	-0.15670300
H	-0.76715600	-1.52950800	-0.52417700
C	3.23457500	-0.50795200	0.06591700
C	2.65928200	0.48913800	0.81349400
C	0.78999500	-0.36924100	0.06252000
S	1.97051300	-1.43635400	-0.71540800
H	3.21515600	1.18854100	1.42945100
N	1.29585700	0.56995800	0.81837800
C	4.63729600	-0.91933000	-0.11918400
O	4.93696900	-2.00059500	-0.61232400
N	5.57159200	0.00825600	0.32571000
H	5.25061600	0.96743900	0.34627100
C	6.96993200	-0.14831100	0.17613800
C	7.70612400	0.82763500	-0.51792500
C	9.08650500	0.74718500	-0.66446300
C	9.75620000	-0.34136100	-0.11176900
C	9.04576700	-1.30799200	0.59567400
C	7.65960700	-1.22803500	0.77038000
H	9.61573200	1.52070200	-1.20867900
H	10.83264600	-0.42527900	-0.22327700
H	9.57694800	-2.14131200	1.04627000
Cl	6.86759600	2.21259200	-1.22323500
C	6.94119200	-2.25799900	1.60211100
H	7.65219000	-2.82180400	2.21116800
H	6.21412600	-1.78402200	2.26973400
H	6.38485900	-2.95305200	0.96691200

Complex 6

C	6.17340600	-1.86586200	0.78468800
C	5.58354900	-0.47433100	0.56039900
N	6.45453300	0.31847200	-0.30723300
C	7.79357600	0.40649100	0.27696900
C	8.38015700	-0.98550600	0.49572000
N	7.50979800	-1.77378300	1.36453000
H	5.52904700	-2.42487700	1.47359900
H	4.60389200	-0.58454200	0.08584900
H	8.43863200	0.96737800	-0.40906500
H	9.36353400	-0.89436500	0.97144700
C	8.06979300	-3.08416100	1.65437000
H	9.04870900	-2.97059000	2.13127200
H	8.20074000	-3.71412500	0.75307400
H	6.18190900	-2.40996300	-0.18087200
H	5.43061600	0.01572500	1.54281400
H	7.78409200	0.94630100	1.24403300
H	8.52821300	-1.47331900	-0.48805700
H	7.41529500	-3.62129900	2.34871400

C	-2.94669700	4.35455400	-0.14525900
C	-3.61494400	3.25226800	0.45559300
N	-1.74697100	4.28675200	-0.67109100
H	-3.46261700	5.31107200	-0.19662100
C	0.47158000	0.75065600	-0.31199600
C	-0.81779500	0.77142400	0.17834100
C	-1.63261000	1.92724200	0.03577900
C	-1.08461300	3.09561500	-0.58157600
C	0.23718300	3.03937800	-1.09329300
C	1.00647400	1.90810400	-0.97464800
H	-1.21807600	-0.09038100	0.69204700
H	0.64277100	3.91899100	-1.57919600
C	-2.97449100	2.00699400	0.52265500
N	-3.61715300	0.92748400	1.12724900
H	-4.33837700	1.18441800	1.79260900
C	-4.23454500	-2.91615300	-0.50942500
C	-4.50381200	-2.66725000	0.83435600
C	-4.28115100	-1.39981000	1.36249700
C	-3.80266800	-0.34469000	0.56190900
C	-3.56723000	-0.61352900	-0.79348200
C	-3.76828800	-1.88276600	-1.33581100
H	-4.87931800	-3.45909700	1.47065000
Cl	-4.63154900	-1.11553500	3.06036800
Cl	-4.50231400	-4.51576600	-1.16479300
O	-3.58123400	-2.07890500	-2.67637100
C	-2.24931800	-2.43818600	-3.05897700
H	-2.25925300	-2.53247800	-4.14568500
H	-1.53166500	-1.66092300	-2.76789300
H	-1.95383900	-3.39561300	-2.61426700
O	1.31941300	-0.30887600	-0.22744600
O	2.24973300	1.86615400	-1.53610900
C	5.92690200	1.64888000	-0.61634700
C	4.62578300	1.63288900	-1.44491000
H	5.77930500	2.25141500	0.30445800
H	6.70378900	2.16523600	-1.18994200
C	3.36829000	1.88965000	-0.62551200
H	4.67540300	2.40193100	-2.22311400
H	4.53771200	0.67202600	-1.96227100
H	3.22938900	1.13643300	0.15532400
H	3.41234900	2.87711800	-0.14455300
C	0.85094000	-1.49309500	0.40796700
H	0.60283300	-1.31196200	1.46069400
H	-0.02944100	-1.89843100	-0.10489000
H	1.66977500	-2.21009900	0.34543300
C	-4.93906100	3.41023200	0.96394700
N	-6.01546800	3.52036000	1.39568500
H	-3.24867500	0.17993600	-1.45866000

Complex 7

C	-1.55126400	-0.74701000	-0.06590800
C	0.75250800	-0.76812500	-0.17276200
C	-0.31158500	-2.73270100	-0.53183300
C	-1.54998200	-2.14005000	-0.31664500
H	-0.26161700	-3.79987900	-0.73737400
C	-5.00601300	0.60529100	0.23016800

C	-6.25170100	0.66072000	-0.38991100
C	-6.46390100	0.12837300	-1.66293600
C	-5.38804300	-0.47433200	-2.31464700
C	-4.13076500	-0.53400400	-1.71609400
C	-3.93145900	-0.00054600	-0.43430000
H	-7.43573600	0.21710900	-2.13395000
S	-7.61689700	1.46139800	0.47975000
O	-8.57178300	1.93266100	-0.54008200
O	-7.02685100	2.39675500	1.45593400
C	-8.46681400	0.13809200	1.44623300
C	-9.09615600	-0.90777800	0.52704900
C	-7.54890800	-0.44661200	2.51859600
H	-8.33144500	-1.50378300	0.01917700
H	-7.12363100	0.33924500	3.14608800
H	-8.12695700	-1.12279400	3.15653600
H	-6.73316700	-1.02503700	2.07354500
H	-9.25503500	0.73722800	1.91945200
H	-9.73619500	-0.43993100	-0.22375200
H	-9.70757300	-1.59049200	1.12563500
H	-4.86976300	1.04662500	1.21161100
H	-3.29749500	-0.97541400	-2.25071300
H	-5.52106100	-0.88131900	-3.31220500
N	-2.67120300	0.01776200	0.20243200
H	-2.40600100	0.91401100	0.59375100
Cl	-2.97708500	-3.15198800	-0.22679900
N	0.84696400	-2.07546300	-0.48198500
N	-0.39450700	-0.08377700	0.01478700
C	5.17176100	-1.82160600	-0.16242100
C	3.78721000	-1.60927600	-0.18845500
C	3.24449700	-0.32416300	-0.09037300
C	4.12716300	0.77027900	0.01900300
C	5.49920800	0.56044500	0.07213000
C	6.05114200	-0.72600200	-0.02232800
H	3.11227900	-2.44825700	-0.28680000
H	6.15424500	1.41265000	0.22475500
N	1.87862000	-0.00829600	-0.04720300
H	1.69883100	0.95616900	0.20192200
O	3.58322200	2.03943600	0.15524500
C	4.68607900	3.40452200	-1.56974100
C	2.32507500	3.84136200	-0.75368100
H	5.10137500	4.07736800	-0.81268500
H	5.42712000	2.63324300	-1.79234300
H	4.50963100	3.97843100	-2.48567600
H	2.12223000	4.46112700	-1.63229000
H	1.38549200	3.37693100	-0.43945500
H	2.67345400	4.49145600	0.05497700
C	5.67178200	-3.24731800	-0.27448800
H	6.29487200	-3.39547100	-1.16468100
H	6.27523200	-3.54462900	0.59070000
H	4.83242200	-3.94354900	-0.34390600
C	9.21208700	1.05042500	0.16837800
C	8.41973300	0.09326700	-0.73881800
C	7.56145800	-0.93358400	0.07267100
C	8.05062000	-0.95437500	1.54467900
C	8.00569900	0.42873200	2.21696000
N	8.34476000	1.49596200	1.25732700
H	10.13897000	0.55893500	0.52001300
H	9.12154000	-0.44234400	-1.38876700

H	7.45901400	-1.66148000	2.13548100
H	6.99792800	0.62170900	2.60424700
H	8.74920500	2.29052800	1.74136100
H	9.52697100	1.92925200	-0.40447000
H	7.76782500	0.67760600	-1.39438900
H	9.08142300	-1.32938500	1.55241900
H	7.77407700	-1.92536600	-0.33374700
H	8.68536000	0.41385500	3.08534400
C	3.37975900	2.79016900	-1.07551600
H	2.99270200	2.09779200	-1.83656400

Complex 8

C	1.57899400	2.47494100	-0.07098000
C	1.23339300	1.11161700	-0.03845300
C	2.24961200	0.14351500	-0.00097700
C	3.57438200	0.57053400	0.00357800
C	3.93468700	1.91433600	-0.02829900
C	2.91271500	2.86455300	-0.06600600
H	0.79609200	3.22913300	-0.10035000
H	1.99370000	-0.90408400	0.02171400
H	4.97701300	2.20846500	-0.02404300
H	3.16239300	3.92084800	-0.09176900
N	-0.13944600	0.81018600	-0.04163400
Br	4.95349800	-0.75598300	0.05697000
C	-0.85979800	-2.68442300	-0.03078300
N	-2.16208700	-2.82119000	-0.03803900
C	-2.87166600	-1.65089000	-0.03735600
C	-2.26345600	-0.36756200	-0.02936300
C	-0.81044800	-0.38790300	-0.03340400
N	-0.14354900	-1.52707400	-0.02974000
H	-0.26168500	-3.59307100	-0.02717000
H	-0.72996800	1.62615500	-0.07021900
C	-4.47876800	0.59948500	-0.01631300
C	-3.08850500	0.76805000	-0.01691100
C	-4.28690200	-1.69356200	-0.03707700
N	-5.06323700	-0.62838800	-0.02407800
H	-2.68703500	1.77801800	-0.00805000
H	-4.76831000	-2.66964800	-0.04213200
N	-5.33174000	1.67695500	-0.03697200
H	-4.92390300	2.56894100	0.19605900
C	-6.76264200	1.53761900	0.18015000
H	-7.00997600	1.19425500	1.19381300
H	-7.23249900	2.50895100	0.00997600
H	-7.17744200	0.81616500	-0.52538500

Complex 9

C	7.84425400	-0.41653800	0.71326200
C	6.96453800	-1.28356300	0.06703700
C	5.69537700	-0.82884100	-0.28209700
C	5.28384500	0.48260000	0.00804500
C	6.19297800	1.32935900	0.66357500
C	7.46771100	0.89092100	1.01495700

H	7.27276200	-2.29619800	-0.16770400
H	5.02196700	-1.49913400	-0.80708100
H	5.89351400	2.33875600	0.92781600
H	8.15786100	1.55153400	1.52757700
Cl	9.44992300	-0.98046600	1.15543600
C	1.53893700	1.97271200	-1.08963300
C	1.56952300	0.63052800	-0.64465100
C	2.81197300	0.11431200	-0.26998800
C	3.92838300	0.95508100	-0.36631500
C	3.74117200	2.27755900	-0.84175800
C	-0.56466400	1.19146500	-1.16355400
C	0.20937500	0.14184100	-0.69342100
H	2.89185000	-0.90112400	0.09967500
H	4.60419500	2.92964900	-0.95173200
H	-0.08023600	3.17637700	-1.75276800
H	-1.62691900	1.22323700	-1.35532400
N	2.56814200	2.80428600	-1.20230900
N	0.22250200	2.28151300	-1.39907000
C	-0.22778400	-1.20072300	-0.33657600
O	0.56106400	-2.07967500	-0.01128000
C	-2.18598600	-2.56377700	-1.19999400
C	-3.51608600	-2.96328900	-1.19537500
C	-4.41540400	-2.34966700	-0.32536900
C	-3.99174400	-1.30575200	0.50581200
C	-2.64863500	-0.91725800	0.43703300
C	-1.70915000	-1.52937200	-0.38724000
H	-3.82919300	-3.76666900	-1.85203400
H	-5.45069500	-2.66446100	-0.28183400
F	-2.25446200	0.09937700	1.25030700
F	-1.32676100	-3.17481600	-2.03258600
N	-4.84584200	-0.67591500	1.44311400
H	-4.37007100	-0.12888300	2.15310700
S	-6.31030100	0.06653500	0.99947000
O	-6.77388500	0.70968600	2.22851000
O	-7.10994100	-0.93167700	0.29005600
C	-5.87584100	1.35558200	-0.20900800
C	-4.97397200	2.45400800	0.35421000
H	-6.84518200	1.74642000	-0.53465100
H	-5.42288300	0.83074200	-1.05581900
C	-4.69638300	3.54552600	-0.68592600
H	-5.45380300	2.88918300	1.23649600
H	-4.02757900	2.01382500	0.68983600
H	-4.04815500	4.32123800	-0.26770100
H	-4.20336300	3.13781700	-1.57597000
H	-5.62295300	4.02784000	-1.01387900

Complex 10

C	-7.34259100	0.93130400	-0.13032900
C	-6.46008900	0.00210300	0.49069500
C	-7.02828900	-1.05609900	1.22250200
C	-8.41097700	-1.14304700	1.33835100
C	-9.25781600	-0.19705700	0.73173000
C	-8.73332700	0.85068100	-0.01652000
C	-5.25269000	1.52571500	-0.66558900

C	-5.11486100	0.41185300	0.13782000
H	-6.40423300	-1.81550800	1.67943900
H	-8.84722900	-1.96071100	1.90450200
H	-10.33393400	-0.29054900	0.84219300
H	-9.38269800	1.57530700	-0.49819900
H	-4.47110200	2.11166600	-1.12541200
N	-6.57450000	1.85071100	-0.82756800
C	-7.09196700	2.96797500	-1.59118300
H	-7.65909200	3.65169700	-0.95009200
H	-6.25662400	3.51440900	-2.03115200
H	-7.74661400	2.62054700	-2.39786000
C	-1.55159800	-0.22011200	0.10526100
N	-1.27627300	-1.10036500	1.08863000
C	-2.33531700	-1.49066700	1.79901900
C	-3.63383100	-1.05236800	1.56537600
C	-3.81351100	-0.14830100	0.50334000
N	-2.76064400	0.26690800	-0.22508000
H	-2.13236700	-2.19243200	2.60799300
H	-4.45098000	-1.36746600	2.19882100
N	-0.50503800	0.26401600	-0.63649000
H	-0.78753300	0.86540800	-1.39780200
C	3.18308800	0.07278600	-0.21348000
C	3.47023700	-1.22860100	-0.67204300
C	2.40501900	-2.04406200	-1.09806900
C	1.09597100	-1.55330500	-1.10010500
C	0.81724100	-0.25450000	-0.62399100
C	1.87420000	0.53512500	-0.17946500
H	2.60119000	-3.05389900	-1.42892000
H	1.67042700	1.53563900	0.18214400
O	0.02379400	-2.26445000	-1.54429900
C	0.22031800	-3.60146900	-1.96813100
H	0.89771600	-3.66020800	-2.83036000
H	0.61445600	-4.22868900	-1.15834100
H	-0.76355100	-3.97079500	-2.26094700
N	4.80046900	-1.64353700	-0.72168100
C	5.15651500	-2.91387000	-1.31639000
H	6.23313100	-2.92571200	-1.50980400
H	4.91677200	-3.78032200	-0.67672000
H	4.64803800	-3.04151400	-2.27592500
N	4.30546600	0.85012400	0.22835300
C	4.67505900	1.93995100	-0.57905400
O	3.87825000	2.39478000	-1.39110100
C	6.03268100	2.54496700	-0.41105100
H	6.70182800	2.11544700	0.32379400
C	5.15900800	-0.00958900	1.08631700
C	5.79692300	-1.08407900	0.19399600
H	4.43111200	-0.52483500	1.73992200
H	6.20254500	-1.89126700	0.82072300
H	6.63396800	-0.64860700	-0.37146600
N	6.11318700	0.72427800	1.90943600
C	5.41971200	1.59462100	2.86274200
C	7.06378600	-0.14245200	2.60317900
H	4.74513500	2.26752300	2.33213000
H	4.82686900	1.02580400	3.60149000
H	6.15674800	2.19655100	3.40124800
H	7.70726100	0.48037700	3.23018700
H	6.57764900	-0.89041200	3.25705200
H	7.70684200	-0.66679800	1.89329800

H	8.05947700	1.40398200	-2.69279400
H	8.57011800	1.02610700	-3.07938300
C	6.38243100	3.58708400	-1.17116400
H	5.68430200	3.99989000	-1.89257600
H	7.36340800	4.04425900	-1.08577200

Complex 11

C	-6.09391900	0.00736100	-0.16768400
C	-4.69654000	0.19579300	0.01529700
C	-4.21969700	1.49715200	0.20484800
C	-5.12635700	2.55330900	0.14855700
C	-6.48533700	2.37117400	-0.07246300
C	-6.98398800	1.07115800	-0.22325600
C	-5.21293600	-2.12005000	-0.06527700
C	-4.08650300	-1.13387300	0.04295200
H	-3.18663800	1.71510700	0.44497200
H	-7.14065100	3.23389800	-0.10887300
H	-8.04646000	0.90489800	-0.37014100
H	-7.27959300	-1.77462000	-0.30993400
F	-4.65511800	3.81127200	0.32542800
N	-6.36449200	-1.35772500	-0.23911300
O	-5.16871700	-3.33989200	-0.03765500
C	-2.81917100	-1.63539700	0.11447500
H	-2.79433600	-2.71920800	0.21639600
C	0.02345200	0.54754300	-0.47458700
C	0.69243200	-0.56898100	0.04009900
C	-0.28503200	-1.56706700	0.33324000
C	-1.53730800	-1.01889600	0.02996900
N	-1.30924100	0.26852400	-0.44993800
H	-2.01263500	0.82327600	-0.91379400
C	-0.02354300	-2.92529800	0.90824800
C	0.52159500	1.84373600	-1.03359500
H	0.15955500	-2.86851500	1.98850300
H	-0.87210400	-3.59733100	0.75725200
H	0.86871000	-3.35814500	0.45425400
H	-0.05680400	2.13681700	-1.91662900
H	0.43990100	2.66465300	-0.30875900
H	1.56891600	1.75844800	-1.32721900
C	2.15949700	-0.75229700	0.20651000
O	2.71102700	-1.84172400	0.05786200
N	2.87645100	0.37370700	0.53331700
H	2.36766300	1.18125400	0.85664800
C	4.31195900	0.30246300	0.79308900
C	5.13558300	0.34455200	-0.50378600
H	4.53858700	-0.62912500	1.31926600
H	4.57181100	1.13886300	1.44850000
H	4.83515200	-0.52094000	-1.10017900
H	4.85779700	1.25048600	-1.07869300
N	6.56801100	0.25678400	-0.24129400
C	7.32938800	-0.27482400	-1.37499900
C	8.71811500	-0.77950800	-0.98237800
H	6.75923700	-1.11281500	-1.78935900
H	7.42874300	0.47105200	-2.18902000
H	9.35732600	0.02501900	-0.60515000
H	8.64105900	-1.54578600	-0.20532800

H	9.22020700	-1.21480600	-1.85276400
C	7.13938800	1.49030600	0.32636800
C	7.71573200	1.30101000	1.73268600
H	6.36729600	2.27245100	0.35395100
H	7.92298600	1.87962300	-0.33990200
H	8.12834100	2.24217900	2.11475100
H	6.94454000	0.95683000	2.42873000
H	8.51299300	0.55266600	1.73133200

Complex 12

C	2.32568800	1.71071500	-0.21311000
C	1.51399100	2.93094400	-0.16534200
C	0.05373600	2.68400000	-0.11956000
C	-0.43991100	1.36109600	-0.06945300
C	1.77603900	0.48249300	-0.17324000
H	3.39671100	1.83117500	-0.30643200
C	-0.90711100	3.72546000	-0.12186000
C	-1.79888300	1.01204300	-0.00963900
C	-2.69458400	2.09096800	-0.00646900
C	-2.26302500	3.42115100	-0.06748100
H	-2.99601300	4.22651000	-0.06394900
O	0.42708500	0.29614600	-0.06841200
O	2.03565100	4.04296700	-0.18465200
O	-4.02680500	1.80357200	0.07207400
O	-0.47958100	5.00876300	-0.17566000
H	-1.25067500	5.59236500	-0.17547800
H	-4.53314000	2.62483300	0.03006500
C	4.26749000	-2.40739200	0.11378900
C	3.64060500	-1.18457500	0.35981800
C	2.48111200	-0.80976500	-0.33984000
C	1.96626700	-1.71598400	-1.28592000
C	2.58789100	-2.93402700	-1.53800800
C	3.74429600	-3.27963700	-0.83729100
H	5.15677800	-2.66786500	0.67636000
H	1.06899800	-1.42985500	-1.82575900
H	2.17551200	-3.60945900	-2.28102000
H	4.23967800	-4.22737300	-1.02419400
Cl	4.33648800	-0.16223700	1.60968100
C	-2.54197100	-2.43948300	1.66185700
C	-2.27625000	-0.91794400	1.58654200
C	-2.34525100	-0.40035800	0.12238800
C	-1.74546000	-1.49316400	-0.77165300
C	-2.68078500	-2.72331800	-0.73972400
N	-3.38097300	-2.85403700	0.53916400
H	-3.06817300	-2.68316000	2.59061700
H	-3.01778100	-0.37298000	2.17835100
H	-3.42550100	-2.60771600	-1.53881000
O	-1.58818900	-1.00446100	-2.10414400
H	-1.36280400	-1.75650800	-2.66848000
H	-2.08246900	-3.62930100	-0.97274200
H	-0.76518500	-1.78068600	-0.37456400
H	-1.29242800	-0.69724600	2.01396400
H	-1.58656500	-3.00029800	1.69115300
C	-3.96932100	-4.17045800	0.70085900
H	-4.55903600	-4.20462400	1.62244200

H	-4.64202300	-4.37920000	-0.13816700
H	-3.22056300	-4.98636500	0.74151800
H	-3.40146400	-0.33843200	-0.14721600

Complex 37

C	5.39912400	0.94575200	0.17416900
C	4.52473800	-0.15800400	0.05200800
C	5.05639100	-1.44598200	-0.01964600
C	6.44054900	-1.58156200	0.03373400
C	7.30486300	-0.49706800	0.15403900
C	6.77780600	0.79822900	0.22630600
C	3.27199000	1.83412400	0.14292900
C	3.15622800	0.35740500	0.02736100
H	4.43663500	-2.33123300	-0.11389100
H	8.37386300	-0.67259900	0.19004000
H	7.43598800	1.65619600	0.32030600
H	4.96687500	3.05182000	0.31179200
F	6.96936000	-2.82569600	-0.03459200
N	4.62253300	2.10820200	0.22506300
O	2.40162800	2.71827900	0.16900400
C	2.01791700	-0.40560000	-0.07954200
H	2.18684100	-1.47792100	-0.14636200
C	0.64717500	-0.05256200	-0.11793100
C	-0.47581800	-0.89961500	-0.21705300
C	-1.61980600	-0.06055100	-0.22293900
C	-1.16915500	1.27333200	-0.11025600
N	0.17602000	1.25137800	-0.05009200
H	0.83295100	2.04283600	0.04644000
C	-0.46762700	-2.39508000	-0.29159300
H	-1.11349600	-2.75045400	-1.09864100
H	0.53888900	-2.78950900	-0.45407800
H	-0.87011100	-2.83122200	0.62737300
C	-1.91254100	2.57065200	-0.02589400
H	-2.85042600	2.44956700	0.52123600
H	-1.30904300	3.32527100	0.48593700
H	-2.15310200	2.97237200	-1.01974100
C	-3.01134500	-0.56910100	-0.28997000
O	-3.30603300	-1.71558600	0.05053500
N	-3.96926800	0.30286700	-0.75145900
H	-3.66774100	1.14138900	-1.22050300
C	-5.35214800	-0.12979200	-0.93067700
C	-6.13617800	-0.10398600	0.39065400
H	-5.36697300	-1.15035000	-1.32347300
H	-5.80813500	0.53022200	-1.67391400
H	-5.63236100	-0.79513300	1.07154700
H	-6.06869600	0.90667700	0.84086200
N	-7.51829900	-0.54548100	0.22172900
C	-8.36572400	0.47445700	-0.38138700
C	-8.09290100	-1.02540800	1.47220600
H	-7.97573300	0.77458400	-1.35806600
H	-9.37064600	0.07104700	-0.54079900
H	-8.45904600	1.38590300	0.24203600
H	-9.09921900	-1.41586900	1.28834200
H	-7.48185600	-1.83945900	1.87267600

H	-8.17259300	-0.24104100	2.25010600

Complex 14

C	1.13833500	-2.17773600	-1.73167200
C	1.39848000	-0.86479200	-1.35263500
C	2.44815800	-0.55999000	-0.46581900
C	3.28480400	-1.62040400	-0.02518400
C	2.98976700	-2.93703600	-0.40603100
C	1.91781700	-3.22808200	-1.24056500
H	0.32384700	-2.37691900	-2.42297100
H	0.78884800	-0.05973600	-1.73597100
H	3.61742300	-3.73187000	-0.01794000
H	1.70215400	-4.25600200	-1.51521600
N	2.71557400	0.75541800	-0.04999900
H	3.59452900	0.83560300	0.47783200
S	4.87418800	-1.40249100	0.85200500
O	4.97236200	-2.57118200	1.74947700
O	4.80904300	-0.02872900	1.43540600
C	6.12470400	-1.45442100	-0.31605800
C	6.62978700	-2.78522200	-0.76816100
C	6.45891200	-0.21932100	-1.08663400
H	6.44552700	-3.56071200	-0.02185700
H	6.17856300	-3.11240300	-1.72344700
H	7.71328800	-2.73503500	-0.94583000
H	6.13131100	0.68485200	-0.57002300
H	7.54662300	-0.14895700	-1.23100100
H	6.01740600	-0.21509500	-2.10031300
C	-0.22502300	2.75544000	-0.05662100
N	0.54739000	1.67334900	-0.17098400
C	1.87105300	1.82077700	0.03307100
C	2.37763800	3.11088700	0.35223800
C	1.47881500	4.15966800	0.41350400
N	0.16345300	4.01195200	0.21308700
H	1.83492500	5.16314900	0.63305300
Cl	4.08058300	3.39182300	0.62884900
N	-1.59420400	2.63656000	-0.22951800
H	-2.06965500	3.52544400	-0.16680700
C	-4.40196300	-0.53373200	-0.40399800
C	-4.79299100	0.77339800	-0.49199500
C	-3.81821200	1.79666800	-0.50942700
C	-2.40591900	1.51429700	-0.33897500
C	-1.99580200	0.20668500	-0.27634800
C	-2.96208200	-0.90512600	-0.48787500
H	-0.94439100	-0.01886200	-0.17200200
O	-4.11260900	3.10802600	-0.60590900
C	-5.46985600	3.52800800	-0.76334600
H	-6.06429700	3.27117100	0.11963400
H	-5.43025800	4.61084400	-0.87572100
H	-5.91645500	3.07836400	-1.65591600
C	-6.19137000	-1.55097000	1.10099400
C	-5.27745200	-1.75557500	-0.15972800
C	-4.20845500	-2.89426000	0.03822900
C	-2.87129000	-2.19789900	0.36320700
H	-4.11307000	-3.44545300	-0.90521700
H	-1.99577200	-2.80106500	0.10979800

H	-2.80225800	-1.93727400	1.42282900
H	-4.50011800	-3.62097600	0.80095800
C	-5.40825800	-1.30793800	2.40002300
C	-7.19574500	-2.69891300	1.29783100
H	-4.69686300	-0.48216600	2.30705100
H	-6.10075200	-1.05804700	3.21012900
H	-4.85482700	-2.19906700	2.71519400
H	-7.79942300	-2.51283300	2.19189700
H	-7.88892200	-2.78909800	0.45729700
H	-6.69544800	-3.66315500	1.44275000
H	-5.84654600	1.02840400	-0.49241000
N	-6.17374000	-1.92294000	-1.32193800
H	-6.65866100	-2.81549900	-1.25703200
H	-5.61835900	-1.96777800	-2.17399200
H	-6.77666200	-0.64843600	0.88421400
H	-2.80139400	-1.25430400	-1.53592100
